# Observation of mirror-odd and mirror-even spin texture in ultrathin epitaxially strained RuO_2_ films

**DOI:** 10.1126/sciadv.aec2917

**Published:** 2026-07-29

**Authors:** Yichen Zhang, Seung Gyo Jeong, Luca Buiarelli, Seungjun Lee, Yucheng Guo, Jiaqin Wen, Hang Li, Sreejith Nair, In Hyeok Choi, Zheng Ren, Ziqin Yue, Jounghoon Hyun, Tieqiong Zhang, Alexei Fedorov, Sung-Kwan Mo, Hojoon Lim, Adrian Hunt, Iradwikanari Waluyo, Junichiro Kono, Ján Minár, Jong Seok Lee, Tony Low, Turan Birol, Rafael M. Fernandes, Milan Radovic, Bharat Jalan, Ming Yi

**Affiliations:** ^1^ Department of Physics and Astronomy, Rice University, Houston, TX 77005, USA.; ^2^ Department of Chemical Engineering and Materials Science, University of Minnesota-Twin Cities, Minneapolis, MN 55455, USA.; ^3^ Department of Electrical and Computer Engineering, University of Minnesota-Twin Cities, Minneapolis, MN 55455, USA.; ^4^ Department of Applied Physics, Kyung Hee University, Yongin 17104, Republic of Korea.; ^5^ Photon Science Division, Paul Scherrer Institute, Villigen 5232, Switzerland.; ^6^ Department of Physics and Photon Science, Gwangju Institute of Science and Technology (GIST), Gwangju 61005, Republic of Korea.; ^7^ Applied Physics Graduate Program, Smalley-Curl Institute, Rice University, Houston, TX 77005, USA.; ^8^ Advanced Light Source, Lawrence Berkeley National Laboratory, Berkeley, CA 94720, USA.; ^9^ National Synchrotron Light Source II, Brookhaven National Laboratory, Upton, NY 11973, USA.; ^10^ Department of Integrative Energy, Myongji University, Yongin 17058, Republic of Korea.; ^11^ Department of Electrical and Computer Engineering, Rice University, Houston, TX 77005, USA.; ^12^ Department of Materials Science and NanoEngineering, Rice University, Houston, TX 77005, USA.; ^13^ Smalley-Curl Institute, Rice University, Houston, TX 77005, USA.; ^14^ New Technologies Research Center, University of West Bohemia, Plzen 301 00, Czech Republic.; ^15^ Department of Physics, The Grainger College of Engineering, University of Illinois Urbana-Champaign, Urbana, IL 61801, USA.; ^16^ Anthony J. Leggett Institute for Condensed Matter Theory, The Grainger College of Engineering, University of Illinois Urbana-Champaign, Urbana, IL 61801, USA.; ^17^ Rice Laboratory for Emergent Magnetic Materials, Rice University, Houston, TX 77005, USA.

## Abstract

Recently, rutile ruthenium dioxide (RuO_2_) has attracted renewed interest due to expectations of prominent altermagnetic spin splitting. However, accumulating experimental evidence suggests that, in its bulk and thick-film forms, RuO_2_ does not display any form of magnetic ordering. Despite this, the spin structure of RuO_2_ remains largely unexplored in the ultrathin limit, where substrate-imposed epitaxial strain can be substantial. Here, we use spin-resolved angle-resolved photoemission spectroscopy, supported by ab initio calculations, to reveal the electronic structure of 2-nanometer-thick epitaxial RuO_2_ heterostructures. We observe an unconventional spin texture characterized by the coexistence of mirror-even and mirror-odd momentum-dependent components. A comprehensive symmetry analysis rules out nonmagnetic origins of this spin texture. These findings suggest an emergent nonrelativistic spin structure enabled by epitaxial strain in the ultrathin limit, marking a distinct departure from the behavior of relaxed or bulk RuO_2_. Our work opens previously unexplored perspectives for exploring symmetry-breaking mechanisms and spin textures in oxide heterostructures.

## Teaser

RuO_2_ in the ultrathin epitaxially strained film form is found to show unconventional momentum-space magnetic spin texture.

## INTRODUCTION

Conventional ferromagnetism and antiferromagnetism are often considered the two archetypal classes of collinear magnetic orders. Although the former displays a net magnetization, in a collinear Néel antiferromagnet, opposite spins are located in sublattices that are related by a lattice translation. However, sublattices with opposite spins can also be related by point-group symmetries of the lattice. Recently, a theoretical classification of collinear magnetic phases based on spin-group theory revealed another type of collinear magnetic order, altermagnetism, where the magnetic sublattices with opposite spins are related by rotations (*1*, *2*). The hallmark of such a fully compensated magnetic order in the electronic spectra is a nodal nonrelativistic spin splitting with d-, g-, or i-wave symmetry (*3*). This is, on symmetry grounds, the same spin splitting realized in metals undergoing a spin-triplet even-parity Pomeranchuk-type instability (*4*, *5*), although altermagnets can also be insulators. Beyond collinear spin arrangements, the spin group formalism has also been recently applied in the classification of noncollinear coplanar and noncoplanar magnetic orders (*6*–*8*), revealing unusual odd-parity magnetic phases (*9*). Overall, the studies of altermagnets and odd-parity magnets revealed a plethora of unique phenomena including electronic topology (*10*–*12*), unconventional spin textures (*6*, *13*–*15*), and chiral responses (*12*, *16*–*18*). Such underlying physics harbored by complex unconventional magnets provides a fertile playground of experimenting with various types of Hall responses (*7*, *19*–*21*) and spin-charge conversion (*14*, *22*), inspiring the development of next-generation spintronics, multiferroics, and thermoelectrics (*2*, *23*–*28*).

Among the predicted altermagnets, RuO_2_ was considered one of the most promising candidates for applications owing to its metallic nature, room-temperature magnetic order, earlier experimental reports supporting magnetism in these compounds (*29*, *30*), and the large energy scale of altermagnetic spin splitting identified in ab initio–based calculations (*2*, *19*, *23*, *31*, *32*). In addition, field-induced nonlinear Hall effect (*33*, *34*), planar Hall effect (*35*), spin-splitting magnetoresistance effect (*36*), spin-splitter torque effect (charge-to-spin) (*37*–*41*), and efficient spin-to-charge conversion (*42*, *43*) were experimentally reported in RuO_2_ films, the origin of which was attributed to the underlying d-wave altermagnetism. Angle-resolved photoemission spectroscopy (ARPES) measurements using magnetic circular dichroism on strain-relaxed RuO_2_ films has reported the observation of time-reversal symmetry (TRS) breaking (*44*, *45*). One spin-resolved ARPES work has reported a d-wave spin texture in RuO_2_ single crystals (*46*). On the other hand, several experimental probes including x-ray diffraction (XRD), unpolarized and polarized neutron diffraction, and muon spin rotation spectroscopy have ruled out the presence of altermagnetism (or any types of magnetism) in both bulk and strain-relaxed film forms of RuO_2_ (*47*–*49*). The absence of altermagnetism in bulk RuO_2_ single crystals was further supported by infrared spectroscopy (*50*), quantum oscillations (*51*), torque magnetometry and magnetization measurements (*52*), as well as Mössbaeur spectroscopy, nuclear forward scattering, and inelastic x-ray and neutron scattering (*53*). Moreover, in the thin film limit, detailed transport examination in ferromagnetic/RuO_2_ heterostructures reported the absence of altermagnetic spin splitting down to 5 nm of RuO_2_ thickness (*54*). This is consistent with the results from time-domain terahertz spectroscopy (*55*). Recent ARPES and spin-resolved ARPES measurements on both single crystals and strain-relaxed films of RuO_2_ have also reached the nonmagnetic conclusion based on measurements of the in-plane spin polarization (*56*, *57*). Theoretical reexamination based on first principles concomitantly revealed the fragile nature and delicate phase boundary of magnetism in RuO_2_ (*58*, *59*).

Despite intense debates and a convergence toward the nonmagnetic nature of RuO_2_ in bulk and thin film forms, the ultrathin epitaxially strained regime of RuO_2_ below a critical thickness of around 4 nm is rarely explored and far from completely understood. Experimentally, it was demonstrated that hybrid molecular beam epitaxy (hMBE)–grown RuO_2_ below 4 nm in RuO_2_/TiO_2_(110) films is fully strained by the substrate along both in-plane directions, reaching −4.7% compressive strain along [001] and +2.3% tensile strain along [1$\bar{1}$0] (*60*). However, in sputter-grown RuO_2_ thin films, metallic behavior has not been reported below this thickness limit. Instead, hMBE-grown RuO_2_ thin films on TiO_2_ (110) substrates exhibit metallic behavior within the fully strained ultrathin thickness regime, attributed to excellent stoichiometry control and atomically smooth interfaces and surfaces (*60*, *61*). Moreover, structural characterization yields a polar structure with point group *mm*2 ($C_{2v}$) (*60*). Rotational anisotropy second-harmonic generation (RA-SHG) and magneto-optical measurements suggested a TRS-broken altermagnetic polar metallic phase in epitaxially strained RuO_2_ with magnetic moments perpendicular to the film direction (*60*, *62*), accompanied by the observation of unusual nonlinear Hall signals at low fields only for the thin and fully strained RuO_2_ layers (*63*). These are in contrast with the observations in thin-to-thick films and single crystals of RuO_2_. In particular, both experimental results and theoretical calculations suggest that epitaxial strain plays a crucial role in stabilizing the magnetic states of RuO_2_ (*60*, *63*, *64*). Therefore, spectroscopy investigation with spin and momentum resolution to resolve the electronic band dispersion of ultrathin epitaxially strained RuO_2_ and its spin character is highly desired.

Here, using spin-resolved ARPES, we probe the momentum-spin-energy-resolved one-electron spectral density of the 2-nm epitaxially strained metallic RuO_2_ films grown by hMBE, a regime of dimensionality and strain that has not been achieved before in spin-resolved ARPES experiments. Narrow bands (NBs) arising from surface states in proximity to the Fermi level modified by the epitaxial strain are observed. Furthermore, we unveil the coexistence of mirror-odd and mirror-even *k*-space spin texture by measuring both in-plane and out-of-plane photoelectron spin polarization. Through symmetry analyses and numerical simulations of the spin-polarized spectra, we find that such observed spin polarization cannot be explained by the polar character of the crystal nor by photoemission extrinsic effects and must point to the intrinsically broken TRS in ultrathin epitaxially strained RuO_2_ films. The possible magnetic point groups are deduced theoretically, consistent with both a ferromagnetic phase and a d-wave altermagnetic phase with magnetic moments pointing within the film plane.

## RESULTS

**Fig. 1.**
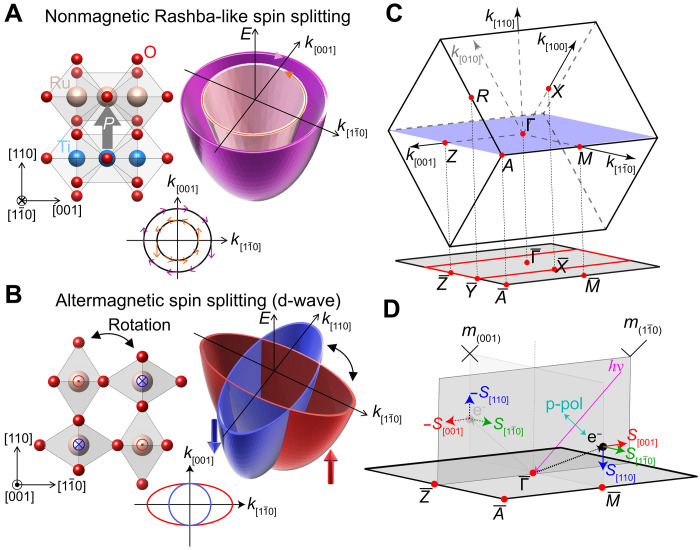
Proposed spin texture relevant in epitaxially strained RuO_2_ and a spin-resolved photoemission measurement geometry. (**A**) Schematic illustration of the charge dipole produced at the (110) RuO_2_/TiO_2_ interface and the associated Rashba-type spin splitting within the (110) plane. (**B**) Decorated local chemical environment of the two Ru sublattices in RuO_2_ related by a *C*_4_ rotational symmetry and the schematic illustration of nonrelativistic altermagnetic spin splitting in *k*-space. (**C**) Bulk Brillouin zone, its projection along [110] (light blue and gray planes), and the (110) surface Brillouin zone (red rectangle) of RuO_2_. (**D**) Schematic illustration of one photoemission geometry used in our experiments, termed as “Geometry A” in Table 1. The (1$\bar{1}$0) mirror of the total photoemission system is indicated as preserved, whereas the (001) mirror is indicated as broken. The three photoelectron spin components are indicated in red, green, and blue arrows.

Given the RuO_2_/TiO_2_ (110) heterostructure, we begin with an illustration of the possible mechanisms that could give rise to spin textures in this geometry. A schematic illustration of the RuO_2_/TiO_2_ interface is given in Fig. 1A, where an electric polarization **P** can be formed along the [110] axis, breaking the inversion symmetry of the system. Consequently, relativistic spin splitting can appear, with an example of the Rashba-type spin texture shown in the plane spanned by the [001] and [1$\bar{1}$0] axes (i.e., the film plane). Such spin texture would show mirror-odd behavior about any vertical mirrors. Meanwhile, if altermagnetism exists between the two Ru sublattices characterized by $\left[C_{2}\|C_{4z}t\right]$ symmetries of the bulk 4/*mmm* point group (here the *z* axis refers to the [001] in bulk geometry and t to a half-translation), as shown in Fig. 1B, a d-wave spin splitting pattern about the [001] axis would emerge, giving rise to spin splitting that is even with respect to the (001) and (1$\bar{1}$0) mirrors. Going from real space to momentum space, we show in Fig. 1C the Brillouin zone of a strained RuO_2_ structure with the [110] *k*-axis pointing along *z* as the experimental out-of-plane direction. Hence, the ARPES in-plane measurements are carried out within the Γ-M-A-Z plane projected to the surface.

An important point that needs to be taken into account in interpreting spin-resolved ARPES measurements is that the combination of incident linearly polarized light and spin-orbit coupling (SOC) can give rise to spin splittings of bands through Rashba and other related effects (*65*). Moreover, extrinsic effects related to the geometry of the experiment and to the detection of photoelectrons may result in an apparent spin-polarized spectrum even when the crystal does not intrinsically break TRS. Multiple scattering of spin-orbit-coupled photoelectrons can produce phase shift and additional spin polarizations in the final states beyond initial states under specific geometries, not only for circularly polarized photons but also under linearly polarized or even unpolarized light (*66*–*73*). To illustrate how these extrinsic, measurement-related sources of spin polarization can affect our measurements, consider our measurement geometry in Fig. 1D (termed as Geometry A), where p-polarized photons shine on a RuO_2_/TiO_2_ (110) heterostructure (structure point group *mm*2) within the (1$\bar{1}$0) mirror plane at off-normal incidence. For an *mm*2 crystal without intrinsic TRS breaking, the detected photoelectron can still exhibit a net spin polarization perpendicular to the preserved mirror $m_{\left(1\bar{1}0\right)}$, which we denote $P_{\left[1\bar{1}0\right]}$ (green) in this case. Here, we use *P* to denote detected photoelectron spin polarization to distinguish from the intrinsic spin polarization of the initial Bloch states, *S*. This means, in this geometry, even the observation of a finite $P_{\left[1\bar{1}0\right]}$ at normal emission (Γ) is allowed without intrinsic TRS breaking of the initial state, as demonstrated in previous studies (*68*, *71*) and confirmed by our ab initio–based one-step model spin-resolved ARPES calculations shown in the Supplementary Text (fig. S9). In contrast, for the two spin components parallel to the (1$\bar{1}$0) mirror ($P_{\left[001\right]}$ and $P_{\left[110\right]}$ colored in red and blue, respectively), the net photoelectron spin polarization cannot be nonzero and must, therefore, reverse its sign after the (1$\bar{1}$0) mirror reflection. Furthermore, because of the beam path and photon polarization depicted in Fig. 1D, the crystal mirror $m_{\left(001\right)}$ is explicitly broken by the measurement geometry. Hence, the even or odd behavior of the photoelectrons with respect to the (001) mirror cannot be inferred in this geometry. Instead, we can use a different geometry (Geometry B) to probe for intrinsic spin texture associated with the $m_{\left(001\right)}$ mirror. A schematic illustration for Geometry B is shown in fig. S8, where the light incidence is rotated azimuthally 90° from Geometry A. The final-state analysis here is reminiscent of the photoemission matrix elements where light with different linear polarizations can be used to probe orbitals with different symmetries even for a system with preserved orbital degeneracy.

To probe for intrinsic broken TRS in the initial state, we first list the expected final-state selection rules in Table 1 based on such analysis for an assumed TRS-invariant *mm*2 state of the epitaxially strained RuO_2_ films measured in Geometry A and Geometry B. We have confirmed that such final-state selection rules numerically using the ab initio–based fully relativistic one-step model approach as shown in fig. S9. Then, any observed photoelectron spin polarization that violates these rules would indicate symmetry breakings beyond extrinsic effects, which, in turn, can be associated with intrinsic TRS breaking under our controlled experiments. In our following spin-resolved ARPES measurements, we carefully choose our measurement geometries, and key observations are made for $P_{\left[001\right]}$ in Geometry A and $P_{\left[110\right]}$ in Geometry B to examine the possibilities of magnetism in the epitaxially strained RuO_2_.

**Table 1.** Extrinsic final-state selection rules for spin-resolved ARPES for our measurement geometry in the nonmagnetic *mm*2 phase of RuO_2_. The light incidence considers p-polarized photons aligned within a mirror plane of the crystal off-normally. The right three columns specify for the nonmagnetic *mm*2 phase of RuO_2_ whether the three orthogonal components of the final-state photoelectron spin polarization (*P*) are allowed to exhibit a finite mirror-even component after being reflected by the preserved mirror plane of the total photoemission system (light + semi-infinite crystals + photoelectrons).

Name	Light incidence	$m_{\left(1\bar{1}0\right)}$	$m_{\left(001\right)}$	$P_{\left[001\right]}$	$P_{\left[1\bar{1}0\right]}$	$P_{\left[110\right]}$
Geometry A	$m_{\left(1\bar{1}0\right)}$	Preserved	Broken	Not allowed	Allowed	Not allowed
Geometry B	$m_{\left(001\right)}$	Broken	Preserved	Allowed	Not allowed	Not allowed

**Fig. 2.**
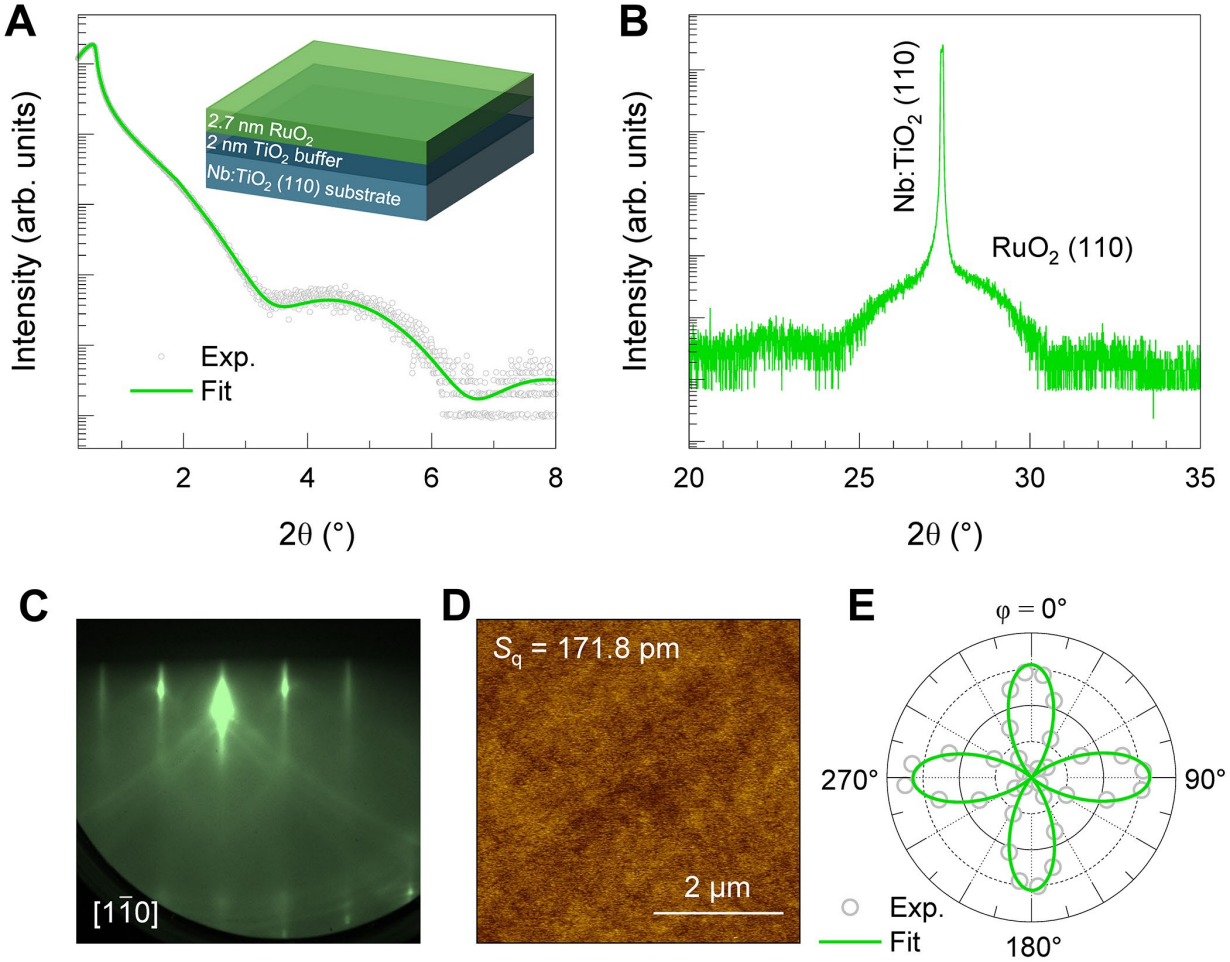
Design and structural characterization of fully strained metallic RuO_2_ (110) heterostructures grown by hMBE. (**A**) XRR and (**B**) XRD 2θ-θ scans of RuO_2_ heterostructures. Scattered symbols and solid lines in (A) represent the experimental data and corresponding fitting results, respectively. The inset in (A) shows a schematic illustration of the heterostructure architecture comprising 2.7-nm RuO_2_/2-nm TiO_2_/Nb:TiO_2_ (110). arb. units, arbitrary units. (**C**) RHEED patterns acquired after growth along the [1$\bar{1}$0] direction reveal streaky features with Kikuchi lines, indicative of high crystalline quality. (**D**) AFM image demonstrating the atomically smooth surface morphology. (**E**) Representative RA-SHG results with both fundamental and SHG polarizations parallel to the incidence plane. Fitting curves (solid lines) based on the noncentrosymmetric *mm*2 symmetry agree well with the experimental data (scattered symbols). Full polarization analysis is included in Supplementary Text.

To experimentally probe the spin texture, we synthesized fully strained metallic RuO_2_ heterostructure via hMBE for spin-resolved ARPES measurements. To avoid charging effects during ARPES measurements, we designed an epitaxial RuO_2_ heterostructure consisting of a nominal 2-nm RuO_2_ layer and a 2-nm TiO_2_ buffer layer grown on a conductive Nb:TiO_2_ (110) substrate, as schematically shown in the inset of Fig. 2A. The conductive substrate effectively eliminated charging effects, whereas the TiO_2_ buffer layer was adopted to suppress unintended interfacial effects due to surface contamination between the Nb:TiO_2_ and the RuO_2_ layer. Figure 2 (A and B) shows the high-resolution x-ray reflectivity (XRR) and XRD θ-2θ scan results, confirming the (110)-oriented out-of-plane lattice structure of the RuO_2_ heterostructures. The XRR data exhibit Kiessig fringes extending to ∼8°, indicating atomically smooth surfaces, and fitting results (solid lines) confirm a RuO_2_ thickness of 2.7 nm (Fig. 2A). XRD θ-2θ scans show clear Laue oscillations around the RuO_2_ (110) Bragg peaks, indicating sharp interfaces. Reflection high-energy electron diffraction (RHEED) images in Fig. 2C taken after growth show streaky RHEED patterns accompanied by the Kikuchi lines along the [1$\bar{1}$0] crystal directions, indicating the excellent crystallinity. Atomic force microscopy (AFM) images in Fig. 2D further reveal an atomically smooth surface with a root mean square roughness ($S_{q}$) of 171.8 pm. In addition, RA-SHG measurements (Fig. 2E) confirm the *mm*2 point group symmetry, indicating a fully strained, noncentrosymmetric RuO_2_ heterostructure (*60*) (see fig. S1 for more details about the SHG analysis). Moreover, to confirm the surface stoichiometry of the RuO_2_ heterostructures grown on TiO_2_ and Nb:TiO_2_ substrates, we have performed ex situ x-ray photoelectron spectroscopy (XPS) (fig. S2), ambient pressure XPS (AP-XPS) (fig. S3), and in situ XPS measurements within the spin-resolved ARPES chamber (fig. S4) to reveal the consistent Ru(IV) oxidation states across samples and the suppression of carbon-related contamination through our in situ oxygen annealing procedures (see Materials and Methods), in agreement with our previous RuO_2_ films (*60*, *74*).

**Fig. 3.**
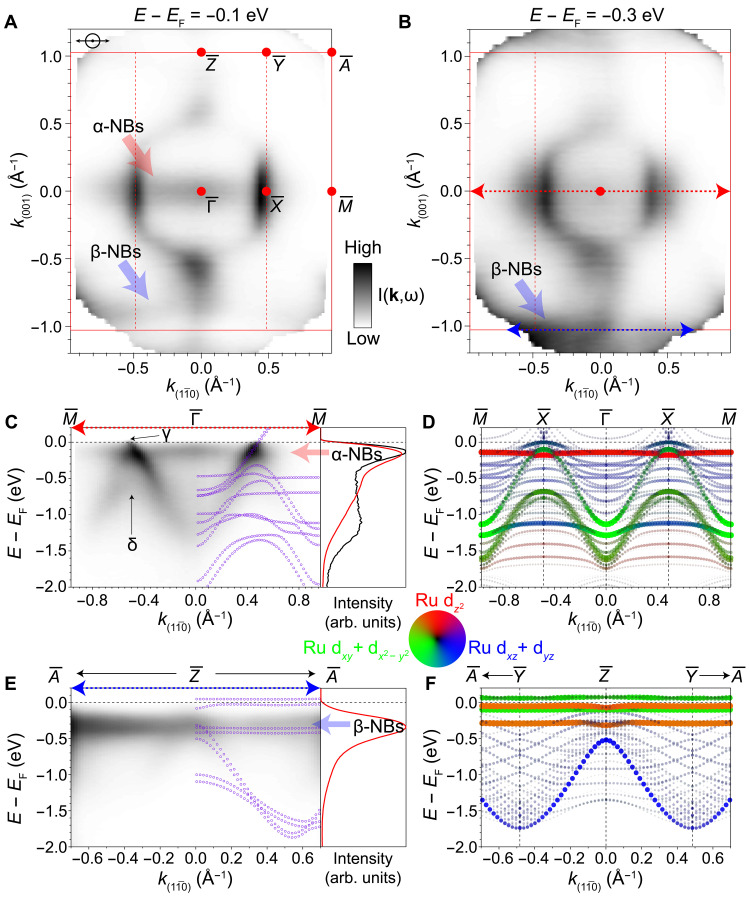
NBs in the ultrathin epitaxially strained RuO_2_. (**A**) CEC at $E-E_{F}$ = −0.1 eV measured by 62-eV p-polarized (indicated on the top left) photons, with an energy integration window of 20 meV, emphasizing the α-NBs and β-NBs denoted by the red and blue arrows. (**B**) CEC at $E-E_{F}$ = −0.3 eV showing the β-NBs. (**C**) Measured electronic band dispersions along the high-symmetry $\bar{\Gamma }-\bar{M}$ direction spanned by the red dotted arrow in (B), overlaid with spin-polarized bulk DFT calculations carried out with full TiO_2_ substrate strain. The EDC integrated across the presented momentum range is shown on the right in red, whereas the black EDC is the single EDC at $\bar{\Gamma }$. (**D**) Non-spin-polarized DFT calculated $\bar{\Gamma }-\bar{M}$ band structure based on a 15-layer inversion-symmetric fully strained RuO_2_ slab (see Supplementary Text for more details). The meaning of the size and transparency of the markers has been specified in the main text. The red, green, and blue colors indicate the projection weights onto the respective $d_{z^{2}}$, $d_{\mathrm{xy}}+d_{x^{2}-y^{2}}$, and $d_{\mathrm{xz}}+d_{\mathrm{yz}}$ orbitals of the Ru atoms within the two surface layers. (**E** and **F**) Same as (C) and (D) but along the high-symmetry $\bar{Z}-\bar{A}$ direction spanned by the blue dotted arrow in (B). All measurements in the main text, unless otherwise specified, were performed at 15 K and with the same p-polarized light. All $k_{x}-k_{y}$ maps in the main text integrate 20 meV in energy.

Before we introduce the observed spin texture, we first present the key features in the electronic structure of the ultrathin epitaxially strained RuO_2_ measured by spin-integrated ARPES. Similar to previously reported band structure of bulk and thick films of RuO_2_ (*56*, *75*–*77*), the Fermi surface (FS) consists of a large $\bar{\Gamma }$-centered hexagonal-like pocket surrounded by smaller pockets, although the fine details differ. However, the most distinguishable spectral features in the ultrathin epitaxially strained RuO_2_ are the NBs near the Fermi level that are largely dispersionless along the [1$\bar{1}$0] axis. Specifically, two sets of NBs appear, one along $\bar{\Gamma }-\bar{M}$ (α-NBs) and one near the zone boundary $\bar{Z}-\bar{A}$ (β-NBs), as can be observed on both constant energy contours (CECs) (Fig. 3, A and B) and high-symmetry momentum slices (Fig. 3, C and E). Both sets of NBs contribute to prominent peaks in the energy distribution curves (EDCs) integrated across the whole momentum ranges (Fig. 3, C and E). The α-NBs also exhibit resolvable wiggling along $\bar{\Gamma }-\bar{M}$, as evident in the distinct peak positions for the single EDC extracted at $\bar{\Gamma }$ compared to the integrated EDC (Fig. 3C). We note that the α-NBs appear at 120 meV below $E_{F}$, distinct from that of the surface NBs observed within 50 meV below $E_{F}$ in cleaved single crystals (*56*, *75*). They are also not observed in our thicker 14-nm films grown by the same methods but under surface annealing at higher temperatures (see Materials and Methods for details) before ARPES measurements (fig. S10) nor present in other strain-relaxed films in the previous literature (*44*, *56*, *76*). Such discrepancy may be attributed to different surface stoichiometries obtained through different postannealing temperatures as the α-NB analogous feature has been shown to disappear on a Ru-rich surface (with oxygen vacancies) (*77*).

To gain insights into the origins of the NBs, we first carried out spin-polarized bulk density functional theory (DFT) calculations. Having explored the effects of the full epitaxial strain and Hubbard *U* correction, we arrived at the best match between calculated and measured dispersions with no *U* but unrelaxed strain where the system hosts a ground state of uncompensated altermagnetism, as shown in Fig. 3 (C and E) (see Supplementary Text and fig. S5 for full details). Notably, the β-NBs observed at about 300 meV below $E_{F}$ in Fig. 3E have been discussed in the previous literature proposing strain-stabilized superconductivity in RuO_2_ with ARPES measurements down to 7 nm where partial strain relaxation exists (*76*). Our bulk DFT calculations confirm such a strain-tuning trend toward $E_{F}$ for the β-NBs and our ARPES data observe it in the fully strained regime of the 2.7-nm RuO_2_ layer. However, the α-NBs are still not captured by these calculations, as shown in Fig. 3C. Therefore, we have carried out DFT slab calculations of RuO_2_ fully strained by TiO_2_ under no Hubbard *U* correction (see figs. S6 and S7 and Supplementary Text). Because of the delicate nature of the magnetic ground states in RuO_2_ slab calculations, we do not rely on them as an indicator for magnetic orders. Therefore, non-spin-polarized calculations are shown in Fig. 3 (D and F) only in assistance of understanding the orbital components of the NBs. As evidenced by the direct comparison between Fig. 3 (C and D), all three major band features (α-NBs, γ, and δ) observed by ARPES are nicely reproduced by the slab calculations after a shift of the DFT $E_{F}$ by −200 meV. In particular, even the wiggling of the α-NBs is captured in Fig. 3D. It is important to emphasize that such an $E_{F}$ adjustment is not a deliberate choice to only fit the α-NBs but a rigid shift to match all the bands, which can be further validated by the β-NB comparison in Fig. 3 (E and F) involving consistent ARPES data, bulk DFT, and slab DFT calculations, after the same $E_{F}$ shift was applied to the slab calculations. To comply with the surface sensitivity of the vacuum ultraviolet ARPES, the marker size and transparency of the slab calculations correspond to the projected weights onto the summed Ru d and O p orbitals within the top two surface layers of RuO_2_. Through further partial charge projection on Ru d orbitals, it is clear that the α-NBs have strong Ru $d_{z^{2}}$ (red) characters near the surface. This feature, combined with the absence of the α-NBs on the bulk calculation, suggests that its origin are the less hybridized surface Ru $d_{z^{2}}$ orbitals (see fig. S7) that “stick out” of the surface. The fact that this orbital points out of plane would naturally explain the weak in-plane dispersion of this band, in agreement with our ARPES observations. Notice that half of the thickness of the constructed slab exceeds 2 nm, which is close to the experimental thickness of our RuO_2_ films. Hence, combining both the experimental observation and theoretical calculations, we conclude that the ARPES observed α-NBs, reminiscent of the NB surface states reported in single crystals, are distinct in several aspects. First, their presence at around $E-E_{F}\approx -120$ meV is an epitaxial-strain-driven behavior, absent in strain-relaxed films and single crystals. Second, by pushing the film thickness close to the surface limit, the α-NBs largely remain across the film (see fig. S7 and Supplementary Text for full details). Third, this band likely arises from surface rather than bulk states. The impact of these NBs on the electronic properties of ultrathin RuO_2_ films, including its possible role in aiding electronic instabilities (*78*), deserves further theoretical investigations that are beyond the scope of our present work. Overall, the spin-integrated ARPES data show excellent agreement with slab DFT calculations assuming stoichiometric terminations, and the valence band behavior at deeper binding energies further supports that the epitaxially strained RuO_2_ films maintain a near-stoichiometric surface (*77*) (see fig. S11 and Supplementary Text). This is fully consistent with the conclusions drawn from the in situ XPS measurements (fig. S4).

**Fig. 4.**
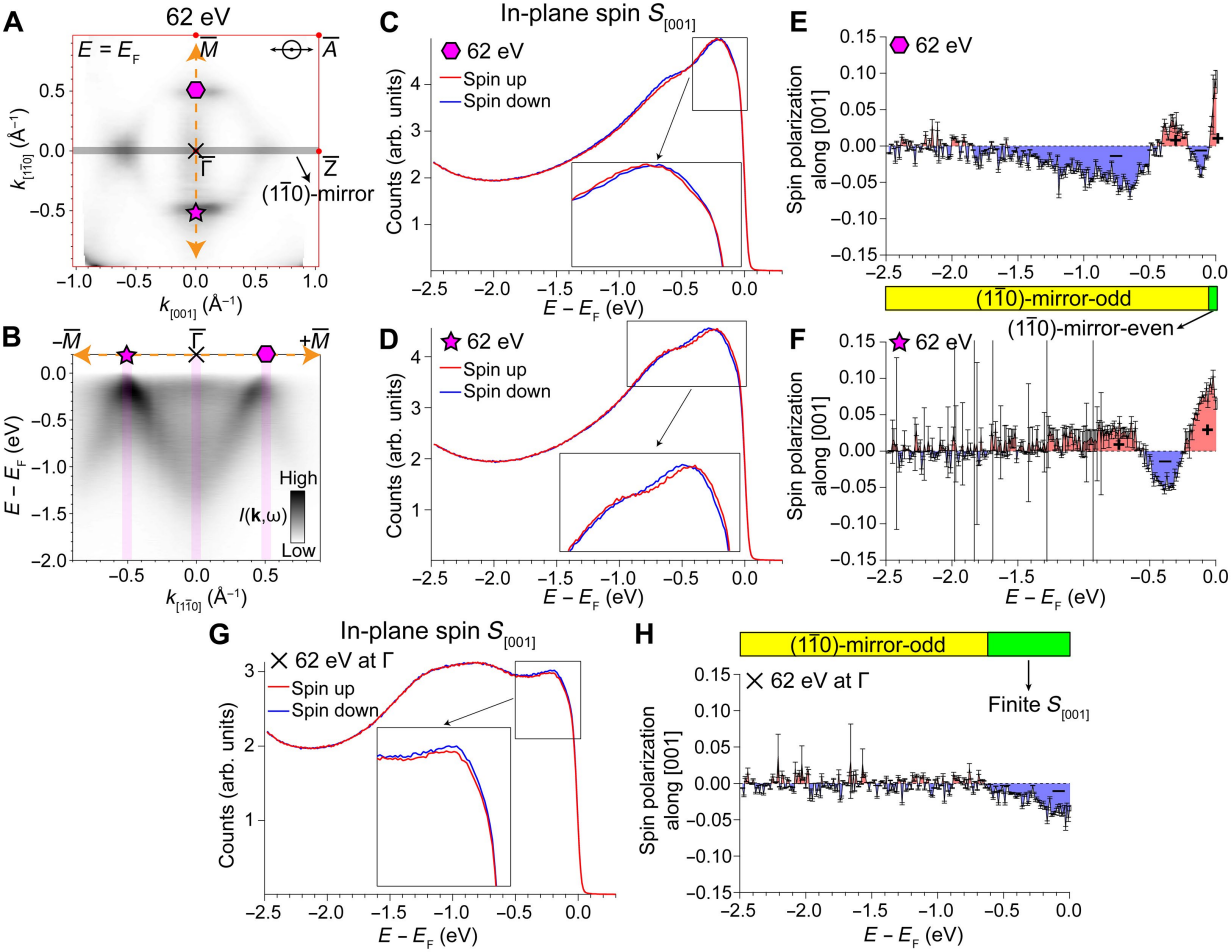
Measured photoelectron spin polarization along the in-plane [001] direction on the $\bar{\Gamma }‐\bar{M}$ path under Geometry A. (**A**) FS probed by 62-eV p-polarized photons (symbol on the top right). (**B**) Band dispersions along the $\bar{\Gamma }-\bar{M}$ direction indicated by the vertical dashed double-headed arrow in (A). The magenta and the cross symbols in (A) and (B) indicate where the spin-resolved EDCs are taken. (**C**, **D**, and **G**) Raw spin-resolved EDCs taken on both sides of the (1$\bar{1}$0) mirror, as well as at $\bar{\Gamma }$, selecting photoelectrons with spin polarization only along the in-plane [001] direction. (**E**, **F**, and **H**) Converted spin polarization based on (C), (D), (G), respectively. See Materials and Methods for details about the background normalization and error bars.

Next, we examine the in-plane spin texture $P_{\left[001\right]}$ with respect to the (1$\bar{1}$0) mirror of the epitaxially strained RuO_2_ film as measured by spin-resolved ARPES. We emphasize that Fig. 4 derives from measurements in Geometry A (see Fig. 1D and Table 1) and the sample was magnetized in situ by an in-plane magnetic field of 0.2 T pointing 45° between the [001] and the [1$\bar{1}$0] film axes. An FS measured by 62-eV photons is shown in Fig. 4A for navigating the spin-resolved EDCs. The measured band dispersions along $\bar{\Gamma }-\bar{M}$ (dashed orange double arrow), which is perpendicular to the mirror, are shown in Fig. 4B. They show similar features to the 62-eV $\bar{\Gamma }-\bar{M}$ data in Fig. 3C measured under Geometry B but differ in terms of intensity asymmetry between $+k_{\left[1\bar{1}0\right]}$ and $-k_{\left[1\bar{1}0\right]}$ due to the photoemission matrix element effects. To probe the spin polarization of bands along $\bar{\Gamma }-\bar{M}$, we measure spin-resolved EDCs at momenta denoted by the magenta and cross symbols in Fig. 4 (A and B). At finite momenta ($k_{\left[1\bar{1}0\right]}\approx \pm 0.5\;{Å}^{-1}$), the measured spin-resolved EDCs are displayed in Fig. 4 (C and D), where spin up and down are respectively represented by red and blue curves and fine details are presented in the zoom-in panels emphasizing the differences between the spin up and down photoelectron counts. Consequently, the derived spin polarization in Fig. 4 (E and F) shows a (1$\bar{1}$0) mirror-odd (flipped spins upon reversing $k_{\left[1\bar{1}0\right]}$) behavior for binding energies away from the Fermi level, consistent with the final-state selection rules in Table 1. However, near the Fermi level, a (1$\bar{1}$0) mirror-even (spins not flipped upon reversing $k_{\left[1\bar{1}0\right]}$) behavior arises, which cannot be explained by the final-state effects of an assumed TRS-preserved state. Furthermore, we directly measure the [001] spin polarization at normal emission ($k_{//}=0$). If the RuO_2_ film had preserved TRS, the $P_{\left[001\right]}$ at Γ would be enforced to be zero according to the selection rules of Table 1. However, as shown in Fig. 4 (G and H), a net spin polarization of $P_{\left[001\right]}$ is observed. This also means that the α-NBs are spin polarized. Photon-energy-dependent counterparts of Fig. 4 (C to H) are presented in figs. S12 and S13 showing a complicated evolution due to multiple scattering of photoelectrons but a consistent symmetry-breaking (1$\bar{1}$0) mirror-even spin polarization component. These observations suggest that the measured epitaxially strained RuO_2_ film exhibits intrinsic magnetic spin polarization, indicating broken TRS. Regarding the out-of-plane spin polarization on the $\bar{\Gamma }-\bar{M}$ path, $P_{\left[110\right]}$, one set of measurements under Geometry B presented in fig. S14 suggests a near-zero amplitude compared to its in-plane components. In addition, measurements on the $\bar{\Gamma }-\bar{M}$ path and along the diagonal in-plane direction for $P_{\left[001\right]}$ are performed in Geometry A, as shown in fig. S15. However, because of the broken (001) mirror of the total photoemission system, we have observed a complicated mixture of (001) mirror-even and odd behavior. The same argument applies as well to the coexisting (1$\bar{1}$0) mirror-odd and even $P_{\left[001\right]}$ spin polarization on the $\bar{\Gamma }-\bar{M}$ path measured alternatively under Geometry B in fig. S16, where the (1$\bar{1}$0) mirror is no longer a preserved mirror of the total photoemission system.

**Fig. 5.**
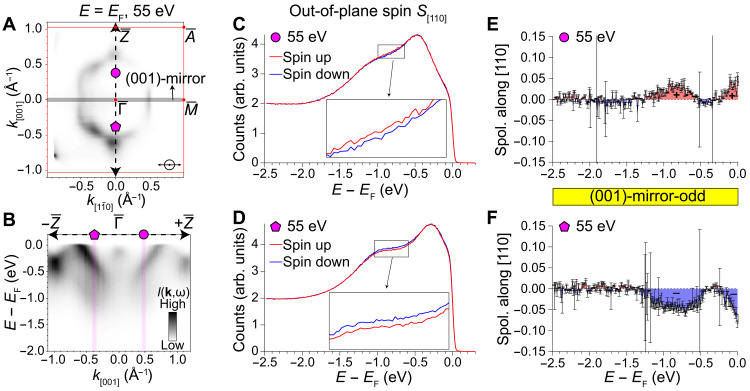
Out-of-plane photoelectron spin polarization on the $\bar{\Gamma }‐\bar{Z}$ path perpendicular to the (001) mirror plane measured in Geometry B. (**A**) FS under 55-eV photons highlighting the (001) mirror and the momentum positions of the measured spin-resolved EDCs using circular and pentagonal symbols. (**B**) Electronic band dispersions measured along $\bar{\Gamma }-\bar{Z}$. Similarly, the width of the vertical magenta bars provides a visualization of the momentum resolution in the spin-resolved measurement mode. (**C** and **D**) Raw spin-resolved EDCs selectively probing only the out-of-plane [110] spin polarization on the upper and lower sides of the (001) mirror. (**E** and **F**) Converted out-of-plane spin polarization from (C) and (D). See Materials and Methods for details about the background normalization and error bar calculation.

Last, we investigate the [110] out-of-plane spin polarization to show that $P_{\left[110\right]}$ channel obeys the *mm*2 TRS-preserving final-state selection rules (Table 1), indicating that intrinsic symmetry breakings are only unambiguously observed for in-plane spins. In Fig. 5, the sample was magnetized in situ by an even stronger out-of-plane magnetic field of 0.4 T and Geometry B was chosen. Nonetheless, as indicated by the selection rules in Table 1, $P_{\left[110\right]}$ would flip sign with respect to the preserved mirror plane of the total photoemission system regardless of Geometry A or Geometry B under the TRS-preserved assumption. In Fig. 5 (A and B), we show the measured FS and $\bar{\Gamma }-\bar{Z}$ band dispersions using 55-eV p-polarized light. The differences compared to ARPES spectra measured at 62 eV in Fig. 3A and fig. S17 (A and B) must be due to kinetic-energy-dependent photoemission matrix element effect rather than $k_{z}$ dispersions as the ultrathin film does not have a well-defined $k_{z}$. Spin-resolved EDCs probing $P_{\left[110\right]}$ at finite momenta with respect to the preserved (001) mirror are presented in Fig. 5 (C and D), and the converted spin polarization in Fig. 5 (E and F). We observe a pure (001) mirror-odd behavior that is compatible within the TRS-preserved final-state selection rules (Table 1). The comprehensive check of the mirror-odd behavior of the out-of-plane spin $P_{\left[110\right]}$ encompassing measurements using different photon energies and at both finite and zero momenta are presented in detail in fig. S17 and Supplementary Text. Besides the out-of-plane $P_{\left[110\right]}$ and the in-plane $P_{\left[001\right]}$, the third spin quantization axis [1$\bar{1}$0] has also been selectively measured under Geometry B and after the same experimental preparation as in Fig. 4, as detailed in the Supplementary Text and fig. S18, showing dominantly (001) mirror-odd behavior but a small even component close to experimental error bars. This violates the final-state selection rules tabulated in Table 1 which enforce $P_{\left[1\bar{1}0\right]}$ to flip sign after the (001) mirror reflection. Therefore, we conclude that the TRS-broken states at 15 K in the 2-nm ultrathin epitaxially strained RuO_2_ films originate from electrons with predominantly in-plane, rather than out-of-plane, spin texture.

## DISCUSSION

Our observations establish a momentum-dependent coexisting mirror-odd and mirror-even *k*-space spin texture, which we associate with broken TRS in the ultrathin epitaxially strained RuO_2_/TiO_2_ (110) heterostructure, after comprehensively ruling out nonmagnetic origins. This is consistent with suggestions from other experimental probes, including SHG, magneto-optic Kerr effect (MOKE), time-resolved MOKE, and Hall transport (*60*, *62*, *63*). In addition, one recent work (*79*) using depth-resolved low-energy muon spin rotation/relaxation measurements suggests a possible reconciliation with nonmagnetic claims of RuO_2_ films of tens of nanometers in thickness, where magnetic signals are claimed to be observed near the surface region. Here, by pushing RuO_2_ films toward 2 nm and the fully epitaxially strained regime, it appears that magnetism can be promoted, consistent with recent polarized neutron reflectometry conclusions (*80*).

The paramagnetic point group *mm*2 of the strained ultrathin RuO_2_ films is polar and, as such, can host unusual topological phenomena. For instance, Kramers nodal lines could exist along the [110] direction, protected by the (001) and (1$\bar{1}$0) mirrors (*81*). Such Kramers nodal lines can support distinct spin textures, as previously reported in SmAlSi and intercalated transition metal dichalcogenides (*82*–*84*). However, in our experiment, $k_{110}$ is not a good quantum number because this is the out-of-plane direction of the ultrathin film.

Although the analysis above compared the experimental data with the final-state spin-resolved ARPES selection rules, we now perform a group-theory analysis to narrow down the magnetic point groups consistent with the observed spin texture. Assuming that the system at high temperatures belongs to the paramagnetic point group *mm*2.1′, we classify the spin-splitting terms that are linear in spin and up to quadratic order in momentum in terms of the irreducible representations (irreps) of the point group (see Table 2; other details are explained in Supplementary Text and table S1). Although symmetry alone cannot predict the magnitude of the spin splittings, it serves as a tool to infer the symmetry properties of the magnetic order parameter. Specifically, we search for the simplest combinations of irreps that give the observed spin-splitting terms in both geometries. In this sense, our analysis gives the highest-symmetry magnetic point group consistent with the data, but lower-symmetry groups cannot be ruled out. In Geometry A, three spin-splitting terms are observed: the *k*-odd term $k_{1\bar{1}0}\sigma_{001}$ and the *k*-even term $k_{1\bar{1}0}^{2}\sigma_{001}$ in Fig. 4 (E and F) as well as the constant $\sigma_{001}$ term in Fig. 4H. The *k*-odd term, according to Table 2, can be ascribed to the $A_{1}^{+}$ irrep, which is to say that it is due to the intrinsic symmetry of the film without the magnetic order. In this notation, the superscript denotes a time-reversal even (+) or odd (−) irrep. In particular, it is a Rashba-like SOC term that is present due to the inversion-breaking effect of the film surface. The *k*-even terms, however, break the TRS and transform as the $B_{1}^{-}$ irrep. We propose that this is the primary magnetic order parameter in our film, consistent with the discussion based on the final-state selection rules. In Table 2, we highlight with underlines the three terms identified in Geometry A. Still, in what concerns Geometry A, fig. S15 (H to I) shows a *k*-even term $k_{001}^{2}\sigma_{001}$ that is also consistent with the $B_{1}^{-}$ irrep, although an odd-in-*k* term inconsistent with this irrep is also seen. We note, however, that the experiment in Geometry A breaks the mirror plane perpendicular to $k_{001}$, which makes the interpretation of these terms less clear. For this reason, we do not highlight these terms in Table 2.

**Table 2.** Classification of constant, even-in-*k*, and odd-in-*k* spin-splitting terms as irreps of the paramagnetic point group *mm*2.1′ up to second order in *k*. Terms dressed with underlines (bold fonts) are those clearly observed in our experiments performed in Geometry A (B). Terms with both underlines and bold fonts were observed in both geometries.

$\mathrm{mm}2.1^{\prime }$	$\sigma_{i}$	$k_{i}\sigma_{j}$	$k_{i}k_{j}\sigma_{h}$
$A_{1}^{-}\left(\mathrm{mm}2.1\right)$	⋅	⋅	$k_{110}k_{1\bar{1}0}\sigma_{001},\;k_{110}k_{001}\sigma_{1\bar{1}0},\;k_{1\bar{1}0}k_{001}\sigma_{110}$
$A_{2}^{-}\left(m^{\prime }m^{\prime }2\right)$	$\sigma_{110}$	⋅	$k_{110}k_{001}\sigma_{001},\;k_{110}k_{1\bar{1}0}\sigma_{1\bar{1}0},\;\left(k_{1\bar{1}0}^{2}-k_{001}^{2}\right)\sigma_{110},\;k_{110}^{2}\sigma_{110}$
$B_{1}^{-}\left(m^{\prime }m2^{\prime }\right)$	$\underline{\sigma_{001}}$	⋅	$k_{110}k_{001}\sigma_{110},\;k_{1\bar{1}0}k_{001}\sigma_{1\bar{1}0},\;\underline{\left(k_{1\bar{1}0}^{2}-k_{001}^{2}\right)\sigma_{001}},\;k_{110}^{2}\sigma_{001}$
$B_{2}^{-}\left(mm^{\prime }2^{\prime }\right)$	$\sigma_{1\bar{1}0}$	⋅	$k_{110}k_{1\bar{1}0}\sigma_{110},\;k_{1\bar{1}0}k_{001}\sigma_{001},\;\left(k_{1\bar{1}0}^{2}-k_{001}^{2}\right)\sigma_{1\bar{1}0},\;k_{110}^{2}\sigma_{1\bar{1}0}$
$A_{1}^{+}\left(\mathrm{mm}2.1^{\prime }\right)$	⋅	$\underline{k_{1\bar{1}0}\sigma_{001}},\;k_{001}\sigma_{1\bar{1}0}$	⋅
$A_{2}^{+}\left(2.1^{\prime }\right)$	⋅	$k_{001}\sigma_{001},\;k_{1\bar{1}0}\sigma_{1\bar{1}0},\;k_{110}\sigma_{110}$	⋅
$B_{1}^{+}\left(m.1^{\prime }\right)$	⋅	$k_{001}\sigma_{110},\;k_{110}\sigma_{001}$	⋅
$B_{2}^{+}\left(m.1^{\prime }\right)$	⋅	$k_{1\bar{1}0}\sigma_{110},\;k_{110}\sigma_{1\bar{1}0}$	⋅

There are spin-splitting terms observed in Geometry B that belong to different irreps than those observed in Geometry A, attesting to the fact that measurements in different geometries can give complementary information. Starting with the spin polarization along the [110] direction, whose corresponding data are shown in Fig. 5 and fig. S17 (E, F, and L), we observe an odd-in-*k* term (but no even-in-*k* or constant term) of the form $k_{001}\sigma_{110}$. This term transforms as the $B_{1}^{+}$ irrep, which breaks the same mirror symmetry as that broken by Geometry B (table S1). Hence, the $k_{001}\sigma_{110}$ term can be interpreted as an SOC effect that does not require TRS breaking. This term is highlighted in bold in Table 2. For measurements taken along the momentum $k_{1\bar{1}0}$ perpendicular to the mirror plane broken explicitly by Geometry B (fig. S14), a negligible spin splitting is observed for the spin component along the [110] direction.

Moving on to the spin polarization along the [001] direction in Geometry B, we observe a constant term $\sigma_{001}$ and an even-in-*k* term $k_{001}^{2}\sigma_{001}$ in fig. S17 (I, J, and N), which transforms as the $B_{1}^{-}$ irrep, as highlighted in bold in Table 2. Although this is a TRS-odd irrep that agrees with the TRS-odd irrep inferred from the measurements in Geometry A, such a term could also be explained by extrinsic final-state ARPES selection rules for Geometry B. Figure S16 (I to L) suggests a combination of even-in-*k* and odd-in-*k* terms $k_{1\bar{1}0}^{2}\sigma_{001}$ and $k_{1\bar{1}0}\sigma_{001}$, which would transform as the irreps $B_{1}^{-}$ and $A_{1}^{+}$, respectively. However, because Geometry B explicitly breaks the mirror perpendicular to $k_{1\bar{1}0}$, these terms are not highlighted in Table 2.

Last, we analyze the data for the $\left(1\bar{1}0\right)$ spin polarization taken in Geometry B. Figure S18 shows a dominant odd-in-*k* term $k_{001}\sigma_{1\bar{1}0}$, which transforms as the trivial irrep $A_{1}^{+}$ and therefore is allowed by the lack of inversion symmetry of the crystal already in the nonmagnetic phase. The same figure also shows smaller contributions from a constant $\sigma_{1\bar{1}0}$ term and an even-in-*k* term $k_{001}^{2}\sigma_{1\bar{1}0}$, both of which transform as the TR-odd irrep $B_{2}^{-}$. This is different from the TR-odd irrep $B_{1}^{-}$ obtained from the analysis of Geometry A, which cannot be explained by the final-state selection rules nor by the combined effect of the primary magnetic order and the explicit breaking of the $m_{\left(1\bar{1}0\right)}$ mirror by Geometry B because $B_{1}^{-}⊗B_{1}^{+}\neq B_{2}^{-}$. It is unclear, from our present investigations, whether this small term reflects another intrinsic magnetic order parameter or is an artifact due to tilting of magnetic moments, existence of minor competing micromagnetic domains, or depolarization fields that might be relevant in ultrathin film geometries. These three terms are also highlighted in bold in Table 2.

Thus, on the basis of the combined final-state selection-rules analysis and intrinsic symmetry analysis, we propose that the observed spin-texture pattern is consistent with the condensation of a primary magnetic order parameter $B_{1}^{-}$ and thus with the magnetic point group *m*′*m*2′, with an additional weaker contribution from $B_{2}^{-}$, further reducing the symmetry to merely 2′. This is consistent both with ferromagnetism and d-wave altermagnetism with in-plane moments. The latter scenario is consistent with the general first-principles expectation that RuO_2_ is close to an altermagnetic instability because, in the presence of SOC, this type of altermagnetic order triggers weak ferromagnetism. We note that this magnetic group is different from that derived from previous SHG experiments in ultrathin films conducted near room temperature instead of 15 K, which found an *m*′*m*′2 magnetic point group (*60*). Although both correspond to d-wave altermagnetism, and could arise from the same spin group in the absence of SOC, in *m*′*m*2′, the moments are in the plane of the film whereas, for *m*′*m*′2, they point out of the plane.

In summary, we have made the following key observations on 2-nm ultrathin epitaxially strained RuO_2_ films: (i) α-NBs and β-NBs under the effect of strain, (ii) mirror-even photoelectron spin polarization pointed along the [001] direction, and (iii) mirror-odd photoelectron spin polarization. Whereas (iii) is a direct consequence of the inversion-symmetry breaking in the RuO_2_/TiO_2_ system, (ii) is unique to the ultrathin epitaxially strained RuO_2_ film not observed in bulk single crystals or thicker films. Their occurrence together here suggests a plausible mechanism where strain plays an essential role in stabilizing the altermagnetic phase, as discussed also in (*60*). It will be interesting to elucidate whether the NBs could also play a role in promoting this instability. Our study therefore reveals important roles played by strain and interfacial effects in RuO_2_ and is instrumental for understanding the debated altermagnetic nature of RuO_2_ and its further spintronic, optoelectronic, and electrocatalysis applications.

## MATERIALS AND METHODS

### Hybrid molecular beam epitaxy

Epitaxial RuO_2_ heterostructures composed of a 2.7-nm RuO_2_ layer and a 2-nm TiO_2_ buffer layer were grown on Nb:TiO_2_ (110) single-crystal substrates (0.5 wt % Nb, Crystec) using an oxide hMBE system (Scienta Omicron). Before growth, the substrates were cleaned with acetone, methanol, and isopropanol, followed by baking at 200°C for 2 hours in the load-lock chamber. Surface treatment was performed with an oxygen plasma anneal at 300°C for 20 min to remove residual contaminants. RuO_2_ layers were grown using a thermally evaporated metal-organic precursor, Ru(acac)_3_, from a low-temperature effusion cell (MBE Komponenten) maintained at 170° to 180°C. The TiO_2_ buffer layer was deposited using titanium tetraisopropoxide (TTIP; 99.999%, Sigma-Aldrich) introduced through a gas inlet system at a beam equivalent pressure of 3 × 10^−7^ torr. Both layers were grown at a substrate temperature of 300°C in an oxygen plasma environment (radio frequency power of 250 W; chamber pressure of 5 × 10^−6^ torr). After growth, the samples were cooled to 120°C in the presence of oxygen plasma to suppress the formation of oxygen vacancies.

### Optical SHG

To characterize the structural symmetry of 2.7-nm RuO_2_/2-nm TiO_2_/Nb:TiO_2_ (110), we conducted RA-SHG measurements. An 800-nm femtosecond pulsed laser with a repetition rate of 80 MHz (VITARA-T, Coherent) was focused onto the sample with a beam size of about 20 μm at an oblique incidence of 45°. The incident fundamental light was set to be either P or S polarized (P_in_ or S_in_), and the second-harmonic light from the sample was obtained in both P or S polarization (P_out_ or S_out_). To isolate the second-harmonic signal, the fundamental light was blocked using a combination of a 450-nm short-pass filter and a 400-nm band-pass filter (Thorlabs). The fundamental light was modulated by a mechanical chopper with a prime number frequency to suppress artifact signals. The modulated SHG signals obtained by the photomultiplier tube (Hammatsu) were demodulated by a lock-in amplifier (SR830, Stanford Research Systems).

### Photoemission spectroscopy

The ARPES and spin-resolved ARPES experiments on the 2-nm RuO_2_/2-nm TiO_2_/Nb:TiO_2_ (110) substrate were performed at the Advanced Light Source beamline 10.0.1.2. After transferring into the ultrahigh vacuum preparation chamber, the film was annealed at 295°C under an oxygen-rich environment of 5 × 10^−6^ torr for 30 min. The oxygen pressure was maintained during the cooling down process toward room temperature. Upon transferring the films into the ARPES chamber and cooling down to a base temperature of around 15 K, a permanent magnet with an in-plane field of 0.2 T was used to magnetize the sample under Geometry A, whereas another permanent magnet with an out-of-plane field of 0.4 T was used to magnetize the sample under Geometry B. A Scienta Omicron DA30L spectrometer was used to analyze the emitted photoelectrons. During the spin-resolved ARPES measurements, VLEED (very-low-energy electron diffraction) detectors were used with the spin quantization axes selectively probing the out-of-plane $P_{\left[110\right]}$ and the in-plane $P_{\left[001\right]}$ and $P_{\left[1\bar{1}0\right]}$ directions. The film was aligned to have negligible angle offsets relative to the analyzer (Γ at $k_{x}=k_{y}=0$) when measured under deflector mode. The spin polarization is calculated from

$$P=\frac{1}{S}\frac{I_{↑}-I_{↓}}{I_{↑}+I_{↓}}$$ (1)

where *S* is the Sherman function taking the value of 0.2 during the time of the measurements. The corresponding spin-up and spin-down EDCs were measured up to the same acquisition time and normalized by the area using the counts within kinetic energies of [55.3, 55.8] eV for data taken under 62-eV light and within kinetic energies of [48.3, 48.8] eV for data taken with 55-eV photon energy. These kinetic energy ranges have been observed to be dominated by the photoemission background signals in the spin-integrated mode and are therefore suitable for the normalization of the spin-resolved EDCs. The error bars of the spin polarization are calculated using the error propagation formula

$$\delta P=P\cdot \sqrt{\frac{{\left(\sqrt{I_{↑}}\right)}^{2}+{\left(\sqrt{I_{↓}}\right)}^{2}}{{\left(I_{↑}+I_{↓}\right)}^{2}}+\frac{{\left(\sqrt{I_{↑}}\right)}^{2}+{\left(\sqrt{I_{↓}}\right)}^{2}}{{\left(I_{↑}-I_{↓}\right)}^{2}}}$$ (2)

where the uncertainty of the spin-resolved photoelectron counts takes the form of $\sqrt{I_{↑}}$ and $\sqrt{I_{↓}}$ assuming the Poisson statistics of $I_{↑}$ and $I_{↓}$.

In situ XPS was carried out inside the spin-resolved ARPES chamber. A pass energy of 10 eV was used to collect the XPS spectra within the kinetic energy range of 20 to 55 eV under 325- and 320-eV photons, where peaks relevant with Ru 3d and C 1s can be covered. Ex situ core-level XPS (Physical Electronics VersaProbe III) were measured with a monochromatic Al Kα x-ray source (1486.6 eV), where a flood gun was used to prevent photoemission-induced surface charge effects.

The ARPES data of 14-nm RuO_2_ on the TiO_2_ substrate were collected at the ULTRA endstation at the Surface/Interface Spectroscopy (SIS) beamline of the Swiss Light Source, Paul Scherrer Institute. The data were acquired with a Scienta Omicron DA30L hemispherical analyzer. The energy and angular resolution are better than 20 meV and 0.1°. The measurements were performed at a temperature of 20 K in a base pressure better than 1 × 10^−10^ torr. The as-received RuO_2_ films were postannealed at 560°C with oxygen pressure 1 × 10^−5^ mbar for 30 min before ARPES measurement.

### Ambient pressure XPS

The AP-XPS measurement was carried out using a synchrotron-based AP-XPS system at the IOS (23-ID-2) beamline of the National Synchrotron Light Source II, Brookhaven National Laboratory (NSLS-II, BNL, USA), which consisted of a differentially pumped electrostatic lens and a hemispherical electron analyzer (Specs GmbH, Phoibos 150 NAP). A focused monochromatic x-ray beam with dimensions of 80 μm (horizontal) by 20 μm (vertical) and a resolving power (*E*/δ*E*) of 10,000 was used (*85*). The sample was annealed under oxygen conditions at 600 K for 30 min to confirm the oxygen annealing effect of RuO_2_ surface bonding states. The base pressure in the analysis chamber was 1 × 10^−9^ torr, and the oxygen pressure was maintained at 100 mtorr during the annealing process. A pyrolytic boron nitride heater was used for annealing, and a K-type thermocouple was attached to the sample surface for temperature monitoring.

### First-principles calculations

The spin-resolved electronic structure of bulk RuO_2_ was calculated by first-principles calculations based on DFT (*86*) as implemented in Vienna ab initio simulation package (VASP) (*87*). We used the projector-augmented wave pseudopotentials (*88*, *89*) and the generalized gradient approximation of Perdew-Burke-Ernzerhof (PBE) (*90*) exchange-correlation (XC) functional. The kinetic energy cutoff for the plane-wave basis was chosen to be 500 eV, and the Brillouin zone was sampled by 20 × 20 × 16 *k*-mesh grid for the primitive unit cell of bulk RuO_2_. To intuitively compare the experimental ARPES results of the RuO_2_ thin film with the theoretical bulk band structure, we plot the band structure of bulk RuO_2_ at a fixed $k_{z}=2\pi /6d$. This specific $k_{z}$ value was selected to effectively describe the quantum confinement effects in the RuO_2_ thin film, using a discretized $k_{z}$ sampling method (*91*).

The inversion-symmetric slab calculations of the strained 15-layer RuO_2_ and 29-layer RuO_2_/TiO_2_ were carried out using the full-potential (linearized) augmented-plane-wave plus local orbitals implementation of DFT (*86*), WIEN2k (*92*). The experimental crystal structure of bulk TiO_2_ was used to construct both the slabs. A vacuum region of 26.2 and 33.9 Å was added into the 15-layer RuO_2_ and 29-layer RuO_2_/TiO_2_ slabs, respectively. The respective slabs have 90 (39 independent) and 174 (74 independent) atoms in the unit cell. All the presented calculations were non-spin-polarized. The structural relaxation was carried out using the generalized gradient approximation (*90*) for the XC functional. All the atomic positions not fixed by the *Pmmm* space group symmetries were allowed to relax. Therefore, out-of-plane partial strain relaxation could be captured in the calculations. Muffin-tin radii of 1.87 $a_{0}$, 1.83 $a_{0}$, and 1.61 $a_{0}$ were chosen for the Ru, Ti, and O atoms, respectively, $a_{0}$ being the Bohr radius. The plane-wave cutoff criteria were set to $G_{\text{max}}$ = 16, $R_{\text{MT}}K_{\text{max}}$ = 6.50 for the 15-layer strained RuO_2_, and $R_{\text{MT}}K_{\text{max}}$ = 6.23 for the 29-layer RuO_2_/TiO_2_. The nonspherical matrix elements were expanded up to *l* = 6. A *k*-mesh of 16 × 7 × 1 was adopted in the full Brillouin zone. The Fermi level ($E_{F}$) was calculated under a Fermi function broadening with a broadening parameter of 0.002 rydbergs (Ry). A simultaneous convergence of energy, charge, and force was required for the convergence of the self-consistent field (SCF) calculations. For the 15-layer strained RuO_2_, it was set to 10^−4^ Ry, 10^−3^ e^−^, and 0.5 millirydbergs per atomic unit (mRy a.u.^−1^). For the 29-layer RuO_2_/TiO_2_, it was set to 10^−4^ Ry, 5 × 10^−3^ e^−^, and 1.0 mRy a.u.^−1^. The charge convergence is only reached when three consecutive SCF iterations are below the charge threshold. A *k*-mesh convergence test was performed for the non-spin-polarized strained 15-layer RuO_2_ using a grid of 32 × 14 × 1 with a Fermi broadening of 0.0018 Ry where a charge convergence of 5 × 10^−4^ e^−^ was achieved. Nonetheless, the relaxed crystal structure and band structure were affected negligibly. The slab calculations use scalar-relativistic approximation, whereas the bulk calculations (purple markers in Fig. 3, C and E) include SOC. Because of the truncated (110) surface Brillouin zone in the slab geometry and without the focus on band unfolding spectral weight, slab band structures along $\bar{X}-\bar{M}$ and $\bar{Y}-\bar{A}$ in Fig. 3 are mirror reflected from $\bar{\Gamma }-\bar{X}$ and $\bar{Z}-\bar{Y}$, respectively.

The one-step model ARPES calculations were performed using the spin-polarized relativistic Korringa-Kohn-Rostoker (SPRKKR) package (*93*), implementing the option of fully relativistic four component Dirac formalism under the atomic sphere approximation. The generalized gradient approximation of PBE (*90*) was used for the XC functional and the crystal structure adopted a WIEN2k relaxed bulk RuO_2_ structure under the strain provided by experimental TiO_2_ lattice constants. We used 32 energy points on the Gaussian-Legendre quadrature path for the energy integration and a *k*-mesh of 17 × 17 × 27 for the Brillouin zone integration during the SCF calculations. The angular momentum summation was cut off at $l_{\text{max}}=4$. The Lloyd’s formula was used for determining the Fermi level. Because the goal is to examine the mirror parities of the photoelectron spin polarization under a paramagnetic assumption in Table 1, we imposed a nonmagnetic constraint during the SCF cycles and no Hubbard *U* correction was used. For the photoemission calculations, the strained bulk crystal was terminated on the stoichiometric (110) surface, using a photoemission Geometry B specified in fig. S8. The final states were modeled as time-reversed low-energy electron diffraction states, and the spin-polarized photoemission intensity was calculated under the spin-density matrix formalism (*94*). Finite lifetime of the initial and final states was simulated with an imaginary potential of 0.01 and 1.5 eV, respectively. The surface potential used a Rundgren-Malmström type *z*-dependent potential (*95*), and both cases of considering and disregarding surface reflections were tested yielding the same mirror-parity conclusions shown in fig. S9.

## Supplementary Materials

### The PDF file includes:

Supplementary Text Figs. S1 to S19 Table S1 Legends for files S1 and S2 References
