# Supplementary material for: Observation of mirror-odd and mirror-even spin texture in ultrathin epitaxially strained RuO2 films
Source: Sci Adv. 2026 Jul 29;12(31):eaec2917. doi: 10.1126/sciadv.aec2917 (PMC13418741; doi:10.1126/sciadv.aec2917)
Supplement: Supplementary file 1 — Supplementary Text Figs. S1 to S19 Table S1 Legends for files S1 and S2 References [file sciadv.aec2917_sm.pdf]

Science Advances
AAAS

Supplementary Materials for

Observation of mirror-odd and mirror-even spin texture in ultrathin epitaxially strained RuO_2 films

Yichen Zhang *et al.*

Corresponding author: Milan Radovic, milan.radovic@psi.ch; Bharat Jalan, bjalan@umn.edu; Ming Yi, mingyi@rice.edu

Sci. Adv. **12**, eaec2917 (2026)
DOI: 10.1126/sciadv.aec2917

The PDF file includes:

Supplementary Text
Figs. S1 to S19
Table S1
Legends for files S1 and S2
References

Other Supplementary Material for this manuscript includes the following:

Files S1 and S2

Supplementary Text

Additional film characterization

Figure S1 presents rotational anisotropy second-harmonic generation (RA-SHG) measurements, consistent with the $mm2$ (C_{2v}) point group symmetry, indicating a fully strained, non-centrosymmetric RuO_2 heterostructure. The RA-SHG patterns in the PP, SP, and PS configurations exhibit a two-fold rotational axis along the [110] direction, with two mirror planes normal to the [$1\bar{1}0$] and [001] directions. The absence of SHG response in the SS configuration, combined with these symmetry elements, confirms that the SHG signal originates from the electric dipole contribution under the non-centrosymmetric $mm2$ point group, where the breaking of the mirror symmetry perpendicular to the [110] axis.

To characterize the surface stoichiometry of the RuO_2 films, x-ray photoelectron spectroscopy (XPS) measurements were carried out under different conditions. First, as shown in Fig. S2, ex-situ Ru $3d$ core-level XPS spectra of RuO_2 heterostructures grown on both TiO_2 and Nb:TiO_2 (with TiO_2 buffer layer) substrates consistently exhibit +4 oxidation states of Ru atoms, confirming the chemical stability across different substrates. However, the presence of carbon contamination in the ex-situ environment is present, as can be seen from the relatively large peak area of the 285 eV binding energy peak relative to the one of the 280.8 eV peak in Fig. S2. Therefore, next we explore the oxygen annealing procedures on the RuO_2/TiO_2 (110) epitaxial films using ambient-pressure-XPS. As shown in Fig. S3 (A and B), annealing under an oxygen pressure of 100 mTorr for 5 min at 600 K strongly suppresses the spectral intensity in the binding-energy region where the Ru $3d_{3/2}$ and C $1s$ signals overlap, indicating effective removal of surface carbon contamination. As a result, the apparent intensity of the Ru $3d_{5/2}$ component becomes dominant, and the intensity ratio between the Ru $3d_{5/2}$ and Ru $3d_{3/2}$ peaks approaches the expected 3:2 spin–orbit branching ratio. This indicates the restoration of a close-to-stoichiometric RuO_2 (110) surface (*96–98*).

Finally, we perform XPS measurements inside the spin-resolved ARPES chamber for the oxygen-annealed 2 nm RuO_2/TiO_2 films. As shown in Fig. S4A, we observe an XPS curve featuring a consistent suppression of carbon contamination and two main peaks belonging to Ru $3d_{5/2}$ and $3d_{3/2}$. We first provide a comparison between XPS data taken at 325 and 320 eV plotted as a function of photoelectron kinetic energy (Fig. S4A), where peaks with a 5 eV shifted kinetic energy

can be confirmed to be core-level peaks, while the 44 eV peak feature in the 325 eV data is strongly suppressed in the 320 eV data and stays at the same kinetic energy. This indicates its kinetic-independent nature and a potential origin from the Auger process. Next, using the 325 eV data, we use a polynomial plus Shirley background and four Lorentzian peaks to fit the curve across the whole measured energy range. We arrived at the best fit with a third-order polynomial background, rather than quadratic, especially for the background at low kinetic energy range. The red (peak 1) and blue (peak 3) peaks are attributed to the Ru $3d_{5/2}$ and $3d_{3/2}$ peaks of RuO_2, the green peak (peak 2) is included for the satellite peak of Ru $3d_{5/2}$ (*99*), while the broad orange peak is used to cover a Ru_{cus} contribution (*98*) under the sharp $3d_{5/2}$ main peak, as well as the 44 eV Auger peak. The extracted peak area ratio between peak 1 and peak 3 is around 1.52, close to a theoretical peak area ratio of 3 : 2 for Ru $3d_{5/2}$ versus Ru $3d_{3/2}$. However, due to the freedom of including more parameters under fitting, such a peak area ratio should not be considered as direct evidence of a perfectly stoichiometric surface. Nonetheless, the in-situ XPS results indicate a similar conclusion that the samples measured by spin-resolved ARPES after the oxygen annealing procedures have suppressed carbon contamination and a close-to-stoichiometric RuO_2 (110) surface.

First-principles calculations on strained RuO_2

To gain insight into the character of the bands seen by ARPES, we perform first-principles calculations (main text Fig. 3 (C and E)), for three distinct sets of parameters for bulk RuO_2: (1) fully-strained without the U correction, (2) unstrained without the U correction, and (3) unstrained with a 2 eV U correction for Ru d orbitals. All these calculations included spin-orbit coupling. Our goal here is not to discuss the possible emergence of altermagnetism in DFT+U calculations, nor the appropriate set of U values that realistically describes the bulk material, but rather to compare the bands obtained in each case with our ARPES data.

As shown in Fig. S5, the set of parameters (3) exhibits the poorest agreement with experimental ARPES (Fig. S5 (C, F, I)), raising doubts on this parameter choice. While the results from the set (2) show slightly better agreement, they still demonstrate limitations, particularly about the narrow band positions (Fig. S5E).

As shown in Fig. S5(A, D, G), the set of parameters (1), corresponding to strained sample without a U correction, shows the best agreement with the experimental results compared to the

two previous cases. Consequently, our results suggest that strain plays a crucial role in determining the magnetic and electronic properties of our thin films of RuO_2.

Further, in order to understand the narrow bands (NBs) along $\bar{\Gamma} - \bar{M}$ (termed as α-NBs in the main text) while being cautious about the error in estimating Fermi energy and magnetic ground states in slab DFT calculations, non-spin-polarized slab calculations in Fig. S6 and Fig. S7 are conducted to approximate the vacuum/RuO_2 and RuO_2/TiO_2 interfaces in experiments. Through our surface annealing procedures described in Materials and Methods of the main text, we are able to obtain a stoichiometric surface termination for the 2.7 nm RuO_2 epitaxial layers in ultrahigh vacuum. Therefore, two types of slab structures with stoichiometric terminations are constructed and compared as shown in Fig. S6 (A and B), a 15-layer RuO_2 slab and a 29-layer RuO_2/TiO_2 slab, both of which possess inversion symmetry, with the layers pointed out by the horizontal arrows containing the inversion centers. Each stoichiometric layer contains two Ru (or Ti) atoms and four oxygen atoms. Under the parameters specified by the Material and Methods section, both the 15-layer RuO_2 and the 29-layer RuO_2/TiO_2 slab structures yield a calculated work function near 5.31 eV. Then the extracted electronic band structure from the two relaxed slabs along high symmetry directions are shown in Fig. S6 (C and D) after projected to orbitals located within the two surface layers. A side-by-side comparison of the two sets of results indicates that the TiO_2 substrate influences negligibly the electronic band structure near the surface region under a model of 7 stoichiometric layers of Ru_2O_4 which has a thickness of roughly 2 nm, close to our ultra-thin film measured experimentally. Therefore, the results from the 15-layer strained RuO_2 slab are chosen to compare with the surface-sensitive ARPES results in the main text. Next we project the band structure to the 7-th layer in Fig. S6 (E and F) to gain insights on effects arising from the RuO_2/TiO_2 interface under an globally inversion-symmetric structure. The 7-th layer corresponds to the RuO_2 layer adjacent to the layer containing the inversion center in the 15-layer RuO_2 structure and the RuO_2 layer interfaced to TiO_2 in the 29-layer RuO_2/TiO_2 structure. Overall, the in-plane Ru d-orbitals (green) are less affected by the TiO_2 substrate, while the out-of-plane Ru d-orbitals (red and blue) show more prominent changes due to the charge transfer to TiO_2. For instance, the d_{z^2} bands dispersing along $\bar{\Gamma} - \bar{X} - \bar{Y}$ roughly from -1.7 eV to -0.2 eV in Fig. S6E show vanishing projected weights in Fig. S6F and the $d_{xz} + d_{yz}$ bands along $\bar{X} - \bar{Y}$ near [-1.7, -1.5] eV relative to the Fermi energy also have altered dispersions. It is worthwhile to point out that the narrow band

features near E_F along $\bar{\Gamma} - \bar{X}$ (half of $\bar{\Gamma} - \bar{M}$) and $\bar{Y} - \bar{Z}$ (half of $\bar{Z} - \bar{A}$) do not present a strong contrast between the 15-layer RuO_2 and the 29-layer RuO_2/TiO_2 results even for the projection onto the 7-th layer.

Next in Fig. S7, we study the site and orbital origins of the $\bar{\Gamma} - \bar{M}$ narrow bands (α-NBs) experimentally observed close to the Fermi level. As shown in Fig. S7A, the strained RuO_2 slab is displayed from the view of [001], denoted as the y-axis, in accordance with the k_y direction adopted in ARPES measurements. The 16 symmetry nonequivalent Ru atoms are denoted using two colors: magenta and black. The magenta ones are the atoms contributing strongly to α-NBs, while the atoms denoted in black provide strong spectral weights for the hole-like dispersive bands topped near the Fermi level at $\bar{X}$, as can be seen from Fig. S7C. In order to emphasize the distinct orbital characters of d_{z^2} and d_{yz} for the α-NBs, the red-green-blue color coding has been divided differently from main text and Fig. S6 so that the three Ru d_{xy}, $d_{x^2-y^2}$, and d_{xz} are grouped into green. Therefore, a clear trend can be observed in Fig. S7B: the α-NBs about 120 meV below the Fermi level consisting of a strong d_{z^2} orbital character of Ru9 at the surface and persisting d_{yz} characters from other Ru atoms marked in magenta extending to the inner layers of the slab.

In the last part of first-principles based calculations, we use the fully relativistic one-step model of ARPES to examine the final state selection rules on the photoelectron spin polarization of an assumed time-reversal-symmetry-preserved RuO_2. As a proof of principle, we perform the calculations in Geometry B, as sketched in Fig. S8, and numerically confirm the results in main text Table. 1. As shown in Fig. S9 (A, C, E), the photoelectron spin polarization defined as $(I_{\text{spin-up}} - I_{\text{spin-down}})/(I_{\text{spin-up}} + I_{\text{spin-down}})$ is projected along the [001], [1$\bar{1}$0], and [110] axes, respectively. We find that the spin-polarizations parallel to the (001)-mirror, $P_{[1\bar{1}0]}$ and $P_{[110]}$, display a (001)-mirror-odd texture, while the polarization perpendicular to the mirror, $P_{[001]}$, shows a (001)-mirror-even spin texture. To further validate such conclusions, we anti-symmetrize $P_{[001]}$ and symmetrize $P_{[1\bar{1}0]}$ and $P_{[110]}$ with respect to the (001)-mirror in Fig. S9 (B, D, F). The results, as expected, demonstrate zero values subject to numerical errors. Notice that the two-dimensional color scale for Fig. S9 (B, D, F) is compressed down to $\pm 1 \times 10^{-3}$.

Additional angle-resolved photoemission spectroscopy measurements in spin-integrated and spin-resolved modes

The angle-resolved photoemission spectroscopy (ARPES) measurements on a partially strain-relaxed 14 nm (110) RuO_2/TiO_2 film have been conducted to visualize its band structure, as shown in Fig. S10. The 14 nm RuO_2 film underwent surface annealing procedure at an elevated temperature of around 560 °C after exposure to atmosphere and before ARPES measurements. The measured Fermi surface is displayed in Fig. S10A. Focusing on the $\Gamma - M$ band dispersions using both linear horizontal and linear vertical polarization of light in Supplementary Fig. S10 (B and C), we do not observe any features associated with the α-NBs, although other dispersive bands exhibit certain resemblance to the data in main text Fig. 2 (C and D). This indicates that the α-NBs could be sensitive to post-annealing temperatures, disappearing due to surface oxygen vacancies at this elevated annealing temperature.

Regarding the fully epitaxially strained 2 nm RuO_2 films, additional ARPES and spin-resolved ARPES measurements have been carried out. In the spin-integrated mode, a $E - k_x - k_y$ mapping has been scanned down to deeper binding energies in Fig. S11 compared to those measured in the main text. Band dispersions along four representative high symmetry directions are extracted to show in Fig. S11 (B to E), where a strong suppression of density of states between 1.7 and 2.4 eV binding energies (indicated by the vertical black double arrows) is observed across the whole data cube. This is an important indication from the valence bands that the measured RuO_2 (110) surface is close to stoichiometry, neither towards oxygen rich, nor oxygen deficient (*77*). According to a detailed study (*77*) on the valence band behavior of single-crystal RuO_2 (110) surface under different surface adsorption conditions combining ARPES and DFT, if the surface were oxygen-rich, along the equivalent in-plane momentum path of $\bar{\Gamma} - \bar{M}$ (see Fig. S11C), a set of valence bands topping around $E - E_F = -2.5$ eV would rise up to $E - E_F = -2$ eV. On the other hand, if the surface were oxygen-deficient, the α-NBs would disappear. However, neither case matches with our observed band dispersions. The persistence of the slightly shifted α-NBs due to epitaxial strain and the strongly suppressed spectral weight between $E - E_F = -2.4$ and -1.7 eV is fully consistent with the stoichiometric surface (*77*).

In the spin-resolved mode, we further investigate the photon energy dependence of the $(1\bar{1}0)$-

mirror-even behavior of $P_{[001]}$ discussed in main text Fig. 4. It is important to note that if the underlying system is time-reversal-symmetry (TRS)-preserved (nonmagnetic or paramagnetic), the final state selection rules imposed by main text Table. 1 apply to all photon energies, regardless of the microscopic details and the multiple scattering of photoelectrons. After reproducing the results of Fig. 4 (C to F) in Fig. S12 (A to C), we also show the $P_{[001]}$ measured at the same in-plane momenta but with photon energies of 55 and 48 eV (Fig. S12 (D to F) and (G to I)). The quantitative spin polarization alters as $h\nu$ varies, while at 55 eV the $(1\bar{1}0)$-mirror-even component near E_F clearly persists. However, at 48 eV the mirror-even component appears dominated by the mirror-odd component. These observations further substantiate the fact that the photoelectron spin polarization does not necessarily reflect the pre-emission spin texture of the Bloch electrons, due to the relativistic effects and multiple scattering effects that must be considered during photoemission. The positive argument is that as long as the final state selection rules are violated and can be observed at certain photon energies, even though not across all photon energies, then there must be symmetry breaking. And in this case, we attribute it to time-reversal-symmetry broken magnetic states. The same argument can be applied to the photon energy dependence of the normal emission finite $P_{[001]}$ shown in Fig. S13.

Next we present the spin-resolved ARPES data measured at momenta that are not perpendicular to the mirror plane of the total photoemission system. First, in Geometry B, the out-of-plane spin polarization $P_{[110]}$ on specific momenta along $\bar{\Gamma} - \bar{M}$ is measured with 55 eV photons, as marked in Fig. S14 (A and B) on both the Fermi surface and $\bar{\Gamma} - \bar{M}$ band dispersion plots. The $(1\bar{1}0)$-mirror indicated by the gray bar in Fig. S14A, although being a symmetry of the RuO_2 film, is no longer a preserved mirror due to the light incidence from right to left on Fig. S14A. The measured [110] spin-resolved energy distribution curves (EDCs) and their associated spin polarization are displayed in Fig. S14 (C to F), showing a negligible value compared to its in-plane spin polarization shown in main text Fig. 4 (E and F). Therefore, it is likely that the initial-state spin polarization near $k_{//} = \pm 0.5$ Å^{-1} on $\bar{\Gamma} - \bar{M}$ is predominantly in-plane. Since the $(1\bar{1}0)$-mirror is not preserved under photoemission, the detected photoelectron spin polarization is strongly affected by the initial-state Bloch electron spin polarization and final-state effects.

Further measured spin-ARPES data along $\bar{\Gamma} - \bar{Z}$, $\bar{\Gamma} - \bar{A}$, and $\bar{\Gamma} - \bar{M}$ are displayed in Fig. S15 (under Geometry A) and Fig. S16 (under Geometry B) for in-plane $P_{[001]}$ spin polarization. As

shown in Fig. S15 (A to C) and Fig. S16 (A to D) the $P_{[001]}$ spin polarization in general shows a mixture of mirror-odd and mirror-even behavior, as evidenced by the datasets in Fig. S15 (H to K) and Fig. S16 (I to L). Similar considerations to those presented above in the context of Fig. S14 apply here, since the (001)-mirror is not preserved in the Geometry A and the $(1\bar{1}0)$-mirror is not preserved in Geometry B. The specific extrinsic mechanisms that determine the photoelectron spin polarization in these cases are beyond the focus of the current work. One final remark for this paragraph is that the relatively large $P_{[001]}$ polarization showcased in Fig. S16 (I to L) at both 55 and 62 eV supports the conclusion of predominant in-plane spin polarization along $\bar{\Gamma}\bar{M}$ near the $k_{//} = \pm 0.5$ Å^{-1} region.

In the following, we present more comprehensive measurements to support the conclusions drawn from main text Fig. 5 measured under Geometry B and with the sample magnetized by an out-of-plane magnetic field of 0.4 T. As shown in Fig. S17A, the (001)-mirror is a preserved mirror under Geometry B. Therefore, along the $\bar{\Gamma} - \bar{Z}$ path, final state selection rules from the second row of main text Table. 1 would apply if the system were TRS-preserved. Experimentally, as shown in Fig. S17 (C to F) and (K and L), we observe the persistent (001)-mirror-odd behavior and a zero spin polarization at normal emission for $P_{[110]}$ at 62 eV, in addition to the consistent observations presented in main text Fig. 5 for 55 eV measurements. Further, we measured the in-plane $P_{[001]}$ spin polarization, which is allowed to be non-zero in this geometry by the ARPES selection rules even if the material does not intrinsically break TRS. As shown in Fig. S17 (G to J) and (M and N), $P_{[001]}$ shows a (001)-mirror-even behavior and a finite spin polarization at normal emission. Therefore, this observed $P_{[001]}$ spin splitting cannot be used as unambiguous evidence of symmetry breaking, unlike the ones carried out in main text Fig. 4 with the sample magnetized by an in-plane field of 0.2 T. These controlled experiments further support the interpretation that the $(1\bar{1}0)$-mirror-even components of $P_{[001]}$ observed in main text Fig. 4 under Geometry A are associated with the intrinsic TRS-breaking occurring within the [001] spin channel.

Finally, we experimentally investigate the photoelectron spin polarization along the third axis, in-plane $[1\bar{1}0]$. To this end, we select the measurement Geometry B, where even the extrinsic effects associated with the ARPES selection rules cannot cause a non-zero net spin polarization $P_{[1\bar{1}0]}$ in the paramagnetic phase. Similar to the experiments for main text Fig. 4, the 2 nm epitaxial RuO_2/TiO_2 film was magnetized by an in-plane magnetic field of 0.2 T pointing 45° between

the [001] and the [$1\bar{1}0$] film axes before the spin-resolved ARPES experiments. The measured Fermi surface and $\bar{\Gamma} - \bar{Z}$ electronic band structure using 62 eV p-polarized photons are shown in Fig. S18 (A and B), where the three momenta along $\bar{\Gamma} - \bar{Z}$ for spin-resolved experiments are marked by magenta and cross symbols. First under 62 eV photons, the spin-resolved EDCs at the three respective momenta are shown in Fig. S18 (C, D, K). At finite momenta, the converted $P_{[1\bar{1}0]}$ exhibits a dominant (001)-mirror-odd behavior in Fig. S18 (E and F). However, an even component also shows up near the Fermi level. This can be also be seen in Fig. S18 (G to J) for the same measurements employing 55 eV photons. At normal emission, both 62 eV and 55 eV data in Fig. S18 (K to N) show a small but finite $P_{[1\bar{1}0]}$, almost on par with the size of the experimental error bars.

Group theory analysis

Bulk single crystal RuO_2 has space group $P4_2/mnm$ (#136). On a single Ru ion, the local site symmetry is mmm (D_{2h}), with generators: m_{001}, $m_{1\bar{1}0}$, m_{110}. The space group further includes the non-symmorphic operations connecting the two Ru ions: $\{4_{001}|(\frac{1}{2}, \frac{1}{2}, \frac{1}{2})\}$ (screw rotation 4_2) and $\{m_{100}|(\frac{1}{2}, \frac{1}{2}, \frac{1}{2})\}$ (glide mirror n). The condensation of dipoles along the [001] direction (B^-_{1g} irrep of mmm) with compensated magnetic order ($m\Gamma^+_2$ irrep of $P4_2/mnm.1'$) yields the magnetic space group $P4'_2/mnm'$. In this group 4_2, $m_{1\bar{1}0}$ and m_{110} must be followed by time-reversal (TR) symmetry. By enforcing the magnetic point group symmetries, we find the allowed spin-splittings for this group (up to quadratic order in $\mathbf{k}$) to be

$$k_x k_y \sigma_z, \tag{S1}$$

$$k_y k_z \sigma_x + k_x k_z \sigma_y, \tag{S2}$$

where we used coordinates x, y, z aligned with the axes [100], [010] and [001]. These are the well known altermagnetic d-wave spin-splittings. The plus sign in Eq. S2 refers to the fact that the splittings $k_y k_z \sigma_x$ and $k_x k_z \sigma_y$ must have the same amplitude, whereas $k_x k_y \sigma_z$ is free to have a different amplitude.

The epitaxial strain ε_{xy} causes the symmetry to lower to $Cmmm$ (#65), where the non-symmorphic operations 4_2 and n are now forbidden. Moreover, for very thin films, the system becomes polar due to the breaking of the mirror m_{110} into 2_{110}. Thus the space group symmetry gets further lowered to $Amm2$ (#38) with point group $mm2$ (C_{2v}). For simplicity of notation, we

now rotate into a coordinate system with axes aligned with the conventional cell: $[1\bar{1}0]$ as the new x axis, [001] as the new y axis and [110] as the new z axis.

We assume that we can describe the magnetic phase of thin-film RuO_2 by starting from the paramagnetic group $mm2.1'$. Because we do not know *a priori* what the primary magnetic order parameter is, we list all of the possible symmetry breaking terms and label them by an irrep of $mm2.1'$. In Table. S1 we show the character table of the point group $mm2$, where for brevity we do not separate the TR odd and TR even irreps. By taking the products of the irreps, we construct the spin splitting terms up to quadratic order in $\mathbf{k}$, which are shown in Table. 2 of the main text. The resultant spin-texture of a possible experiment-informed magnetic point group $m'm2'$ is visualized in Fig. S19.

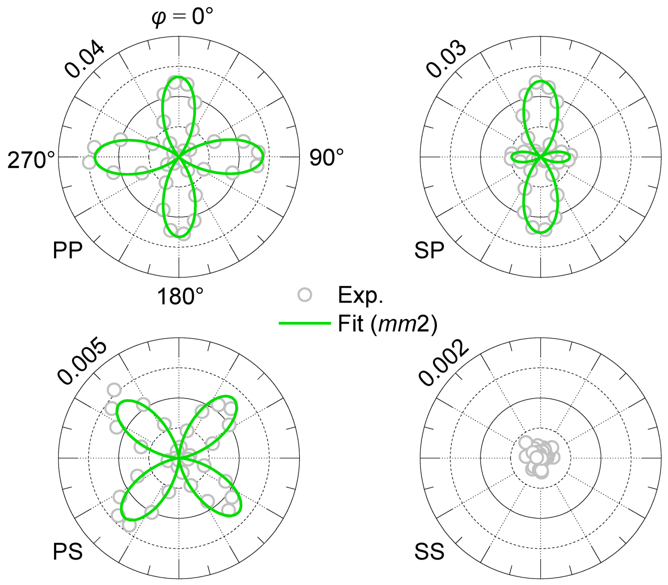

Figure S1: **Rotational anisotropy second-harmonic generation (SHG) results for RuO_2 heterostructures.** SHG intensity was measured as a function of azimuthal angle ϕ under an oblique incidence of 45° at room temperature. The incident fundamental light was set to either P- or S-polarization (P_{in} or S_{in}), and the SHG signal was detected in both P- and S-polarizations (P_{out} or S_{out}), where P and S denote polarization parallel and perpendicular to the plane of incidence, respectively. The fitting curves (solid lines), based on a non-centrosymmetric *mm*2 point group with electric-dipole allowed SHG, show excellent agreement with the experimental data (scattered symbols). A detailed symmetry analysis can be found in our previous study (*60*).

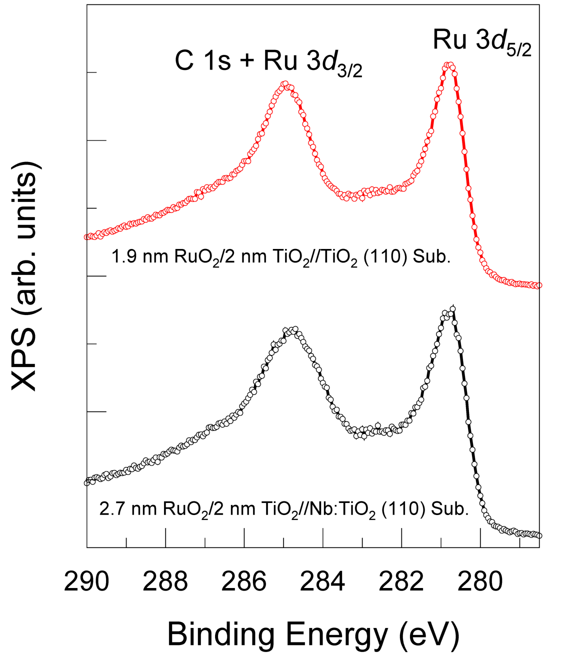

Figure S2: **Ru 3*d* x-ray photoelectron spectroscopy (XPS) spectra of RuO_2 heterostructures grown on TiO_2 and Nb:TiO_2 substrates.** Although the Ru $3d_{3/2}$ peak overlaps with the C 1*s* peak due to their close binding energies, the consistent Ru $3d_{5/2}$ peak position and width in both samples confirm the consistent oxidation state. These are also consistent our previous XPS study of RuO_2 samples (*74*).

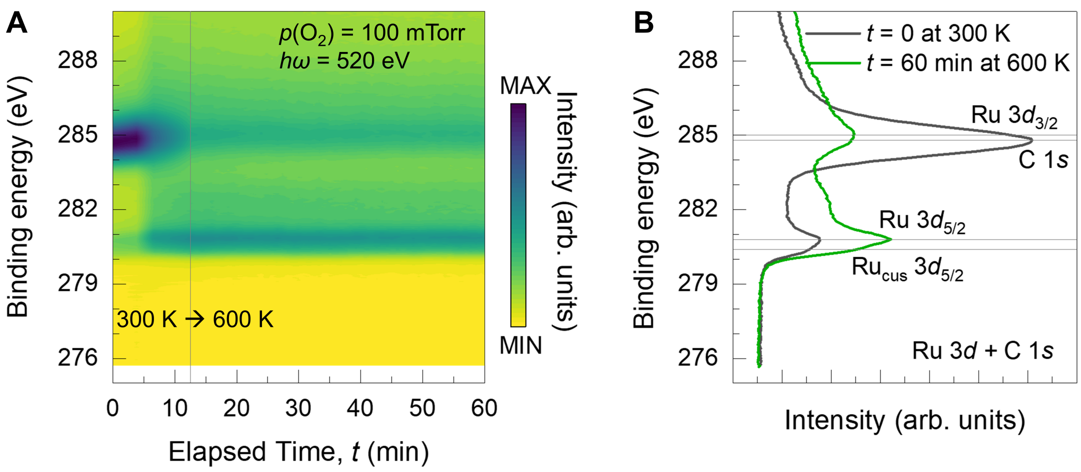

Figure S3: **Oxygen annealing study of RuO_2/TiO_2 (110) epitaxial thin films with AP-XPS.** (**A**) Contour plots of APXPS spectra at Ru 3d and C 1s core levels as a function of elapsed time (t) and binding energy during oxygen annealing process. We have shipped samples to APXPS facilities and loaded UHV chamber. Subsequently, we have annealed sample at 600 K and 100 mTorr of $p(O_2)$ in APXPS chamber; the temperature reached 600 K at t = 13 min, approximately. Acquisition time of each spectrum is 43 seconds, and photon energy ($h\omega$) is used to 520 eV. (**B**) APXPS spectra at t = 0 and 60 minutes. Upon oxygen annealing, the C 1s intensity is strongly suppressed, while the Ru 3d intensity increases, indicating effective surface cleaning. Notably, the binding energy position of the Ru $3d_{5/2}$ peak remains identical, demonstrating that the Ru-O chemical bonding is well preserved during annealing. In addition, a shoulder feature corresponding to coordinatively unsaturated Ru atoms (Ru_{cus} $3d_{5/2}$), previously reported in XPS studies (*98*), is observed, suggesting the presence of Ru_{cus} sites at the surface.

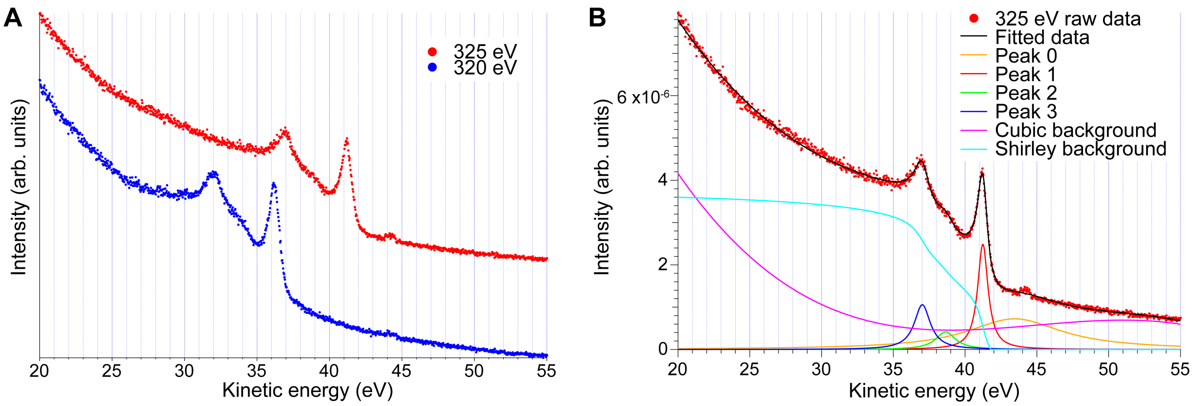

Figure S4: **In-situ x-ray photoelectron spectroscopy (XPS) of the 2 nm RuO_2/TiO_2 epitaxial film measured by spin-resolved angle-resolved photoemission experiments.** (**A**) Raw XPS data targeting Ru 3*d* and C 1*s* binding energy ranges under 325 eV and 320 eV photons. (**B**) The fitting results of the 325 eV data involving Lorentzian peaks, Shirley background, and a polynomial background up to the third order.

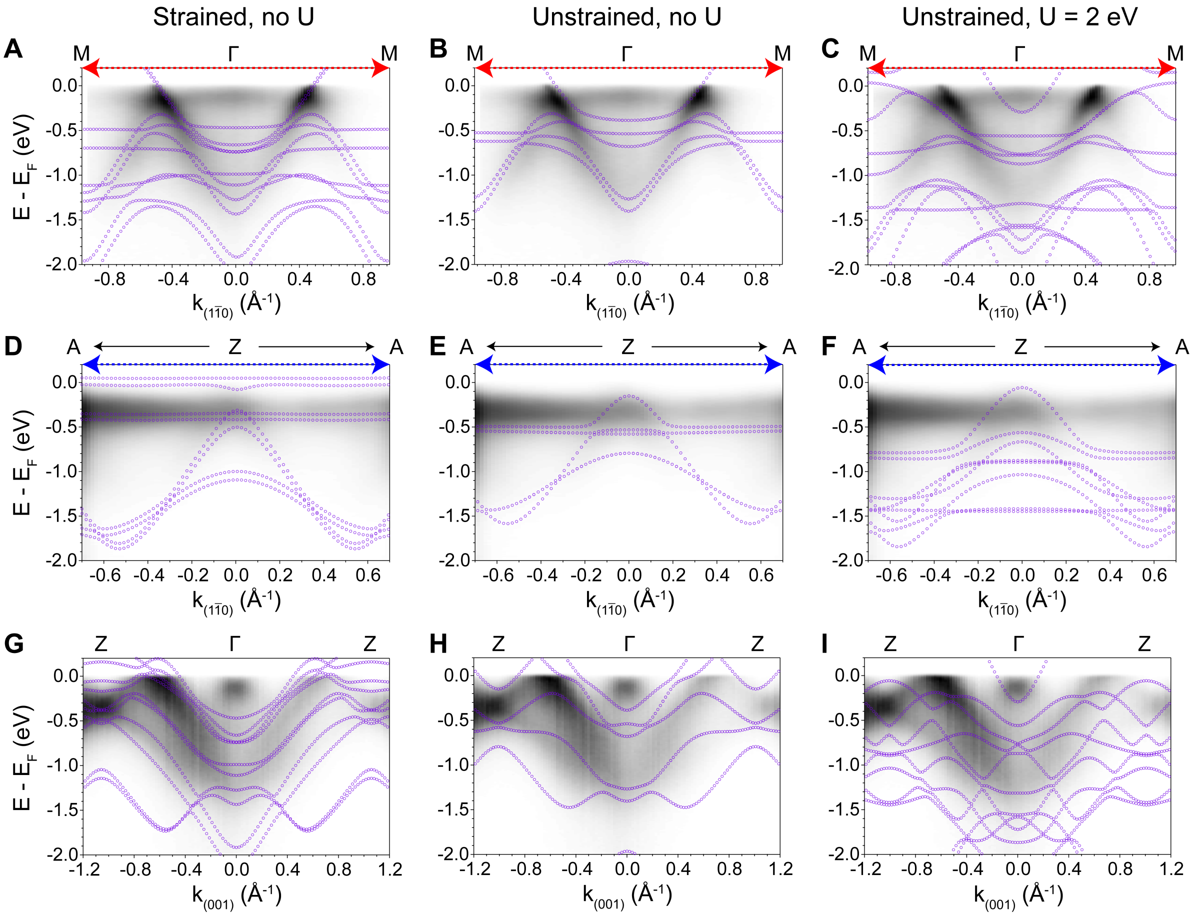

Figure S5: **Comparison of calculated strain- and Hubbard U-dependent electronic structures of RuO_2 with experimental ARPES spectra** (**A**) Calculated electronic structures of bulk RuO_2 (purple dashed line) and ARPES spectra of the 2nm RuO_2 thin film along (**A-C**) M-Γ-M, (**D-F**) A-Z-A, (**G-I**) Z-Γ-Z high-symmetry lines. The bulk RuO_2 electronic structures were obtained using three distinct approaches: (**A**, **D**, **G**) fully-strained without Hubbard U correction, (**B**, **E**, **H**) unstrained without Hubbard U correction, and (**C**, **F**, **I**) unstrained with a 2 eV Hubbard U correction applied to the Ru d orbital. For each case, the self-consistent field ground state is denoted at the top.

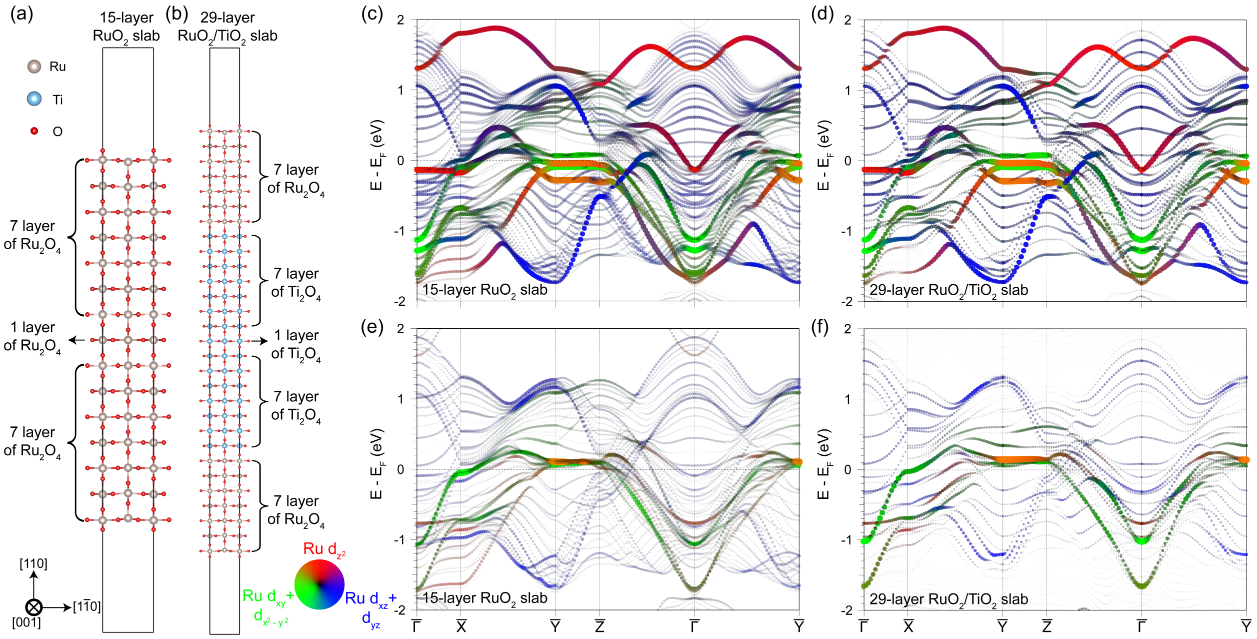

Figure S6: **Non-spin-polarized density functional theory slab calculations for the inversion symmetric strained RuO_2 and RuO_2/TiO_2 heterostructure.** (**A** and **B**) Side views along [001] of the 15-layer strained RuO_2 and the 29-layer RuO_2/TiO_2 slab structures, respectively. The inversion center is contained in the middle layer of RuO_2 or TiO_2. (**C**) Electronic band structure of the 15-layer RuO_2 under the TiO_2 substrate strain where the meaning of the size, transparency, and color-coding of the markers is the same as that of the main text Fig. 3 (D and F). The Brillouin zone notations follow from Fig. 1C. (**D**) Same as (C) but for the 29-layer RuO_2/TiO_2 with the same projection to the surface orbitals. (**E** and **F**) Same as (C and D), but the size and transparency of the markers are projected to the summed Ru d and O p orbitals within the 7-th layer counting from the outmost surface layer as the first layer. The red, green, and blue colors also represent the projection to the combinations of the cubic harmonics of the Ru d orbitals within the 7-th layer. The same E_F shift with Fig. 3 is applied here.

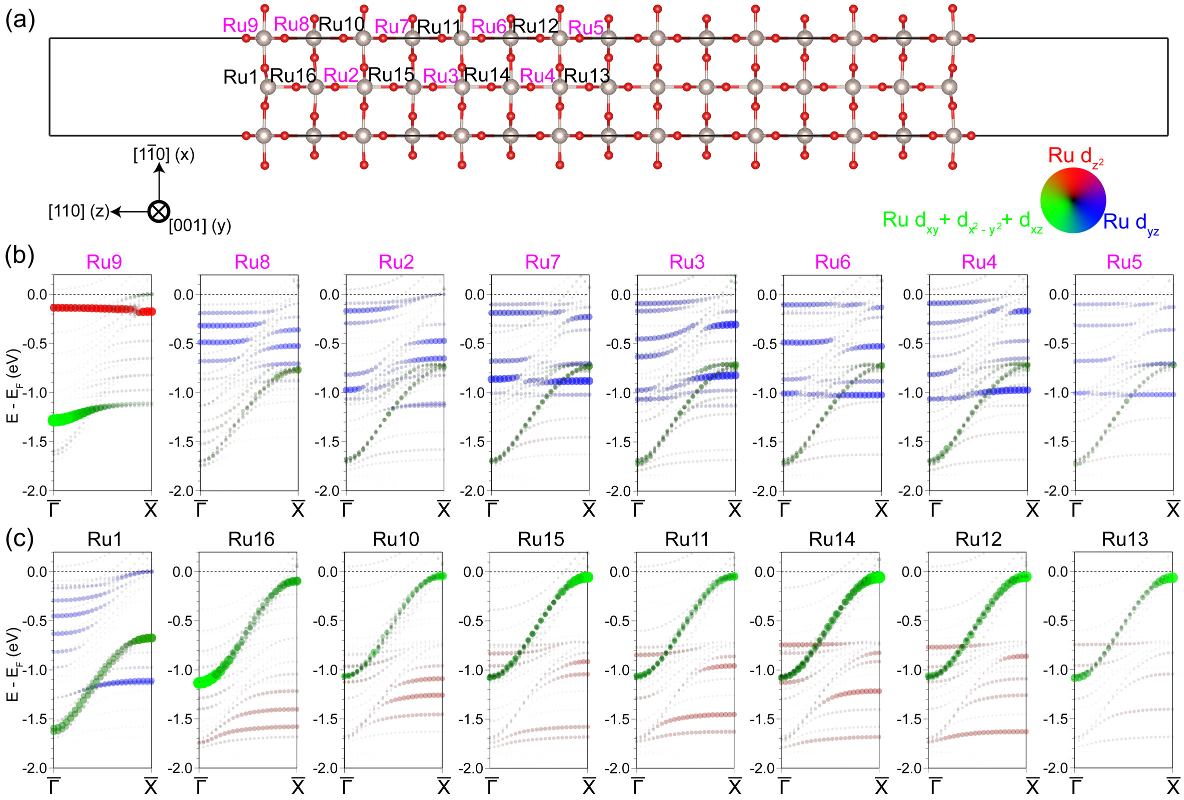

Figure S7: **Site and orbital-resolved electronic band dispersions of the 15-layer strained RuO_2 extracted from non-spin-polarized slab density functional theory calculations.** (**A**) View of the RuO_2 strained slab structure along [001] with numbering on the Ru atoms. The magenta fonts denote Ru atoms that are main contributors to the $\bar{\Gamma} - \bar{M}$ narrow bands near the Fermi level. (**B**) Orbital-resolved band structure along $\bar{\Gamma} - \bar{X}$ (half of $\bar{\Gamma} - \bar{M}$) projected to the d-orbitals of the specified Ru atoms where the red, green, and blue encode the d_{z^2}, $d_{xy} + d_{x^2-y^2} + d_{xz}$, and d_{yz} weights of the single Ru atom, respectively. Both the size and transparency of the markers are proportional to the total projection weights onto the specific Ru atoms. (**C**) Same as (B) but for Ru atoms that do not strongly contribute to α-NBs. The same E_F shift with Fig. 3 is inherited here.

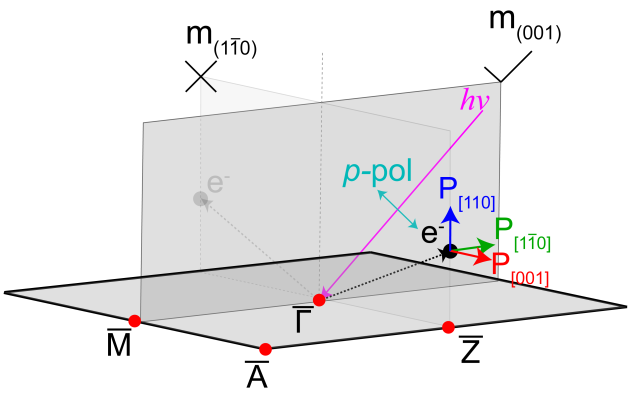

Figure S8: **Schematic illustration of the photoemission Geometry B.** Similar to Fig. 1D but with the beam incidence rotated towards within the (001)-mirror plane, therefore preserving the (001)-mirror but breaking the (1$\bar{1}$0)-mirror of the total photoemission system. The three photoelectron spin components are indicated in red, green, and blue arrows.

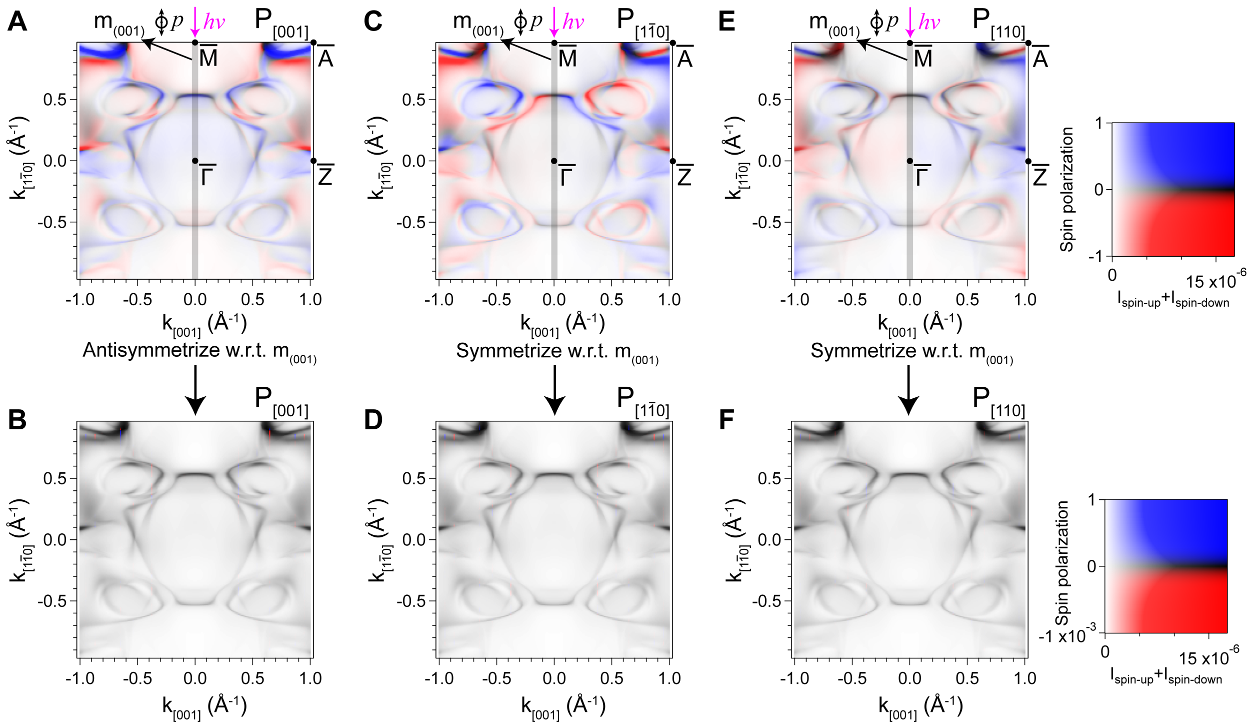

Figure S9: **Photoelectron spin polarization from fully relativistic first-principles calculations using the one-step model of photoemission on the (110) cleaving surface of nonmagnetic RuO_2 under Geometry B and 62 eV *p*-polarized photons.** (**A**) Calculated photoelectron spin polarization on the Fermi surface projected to the [001] crystalline axis ($P_{[001]}$). Vertical gray bar indicates the preserved (001)-mirror. Light incidence with *p*-polarized photons is indicated on the top, fully within the (001)-mirror. A two-dimensional color scale shown on the right of the top row is employed to faithfully visualize the calculated spin texture. (**B**) Anti-symmetrizing the results in panel (A) with respect to (w.r.t.) the preserved (001)-mirror. Notice that the corresponding color scale of the spin polarization is zoomed in to 1×10^{-3}, as indicated on the right of the bottom row. (**C** and **D**) Same as (A and B) but for the photoelectron spin polarization along the $[1\bar{1}0]$ direction. (D) symmetrizes the data in (C) w.r.t. the (001)-mirror. (**E** and **F**) Same as (A and B) but for the photoelectron spin polarization along the [110] axis. (F) symmetrizes the data in (E) w.r.t. the (001)-mirror.

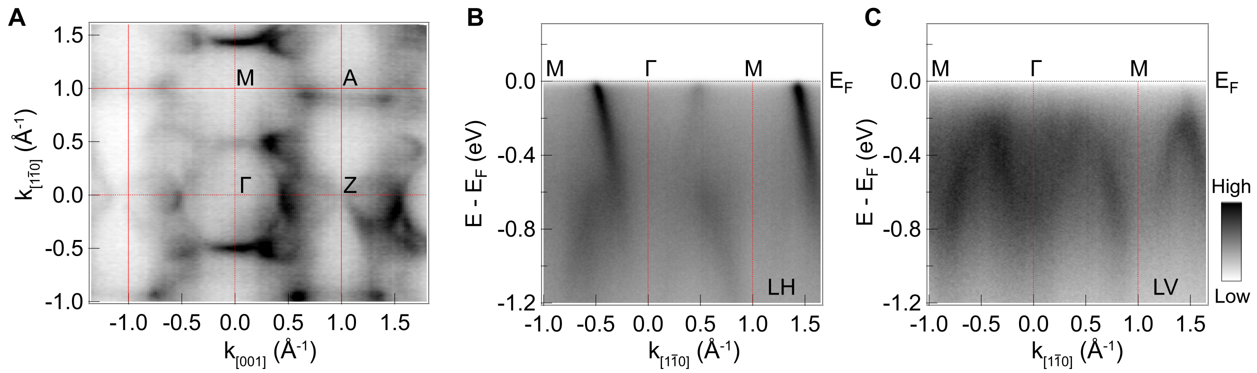

Figure S10: **Measured electronic band structure of the 14 nm strain-relaxed RuO_2 film.** (**A**) Fermi surface of the (110)-plane. **(B)** $\Gamma - M$ band dispersions measured with linear horizontal (LH) polarization. (**C**) $\Gamma - M$ band dispersions measured with linear vertical (LV) polarization. Photons of 130 eV was determined and adopted to measure near the $k_z = 0$ plane in the thicker film.

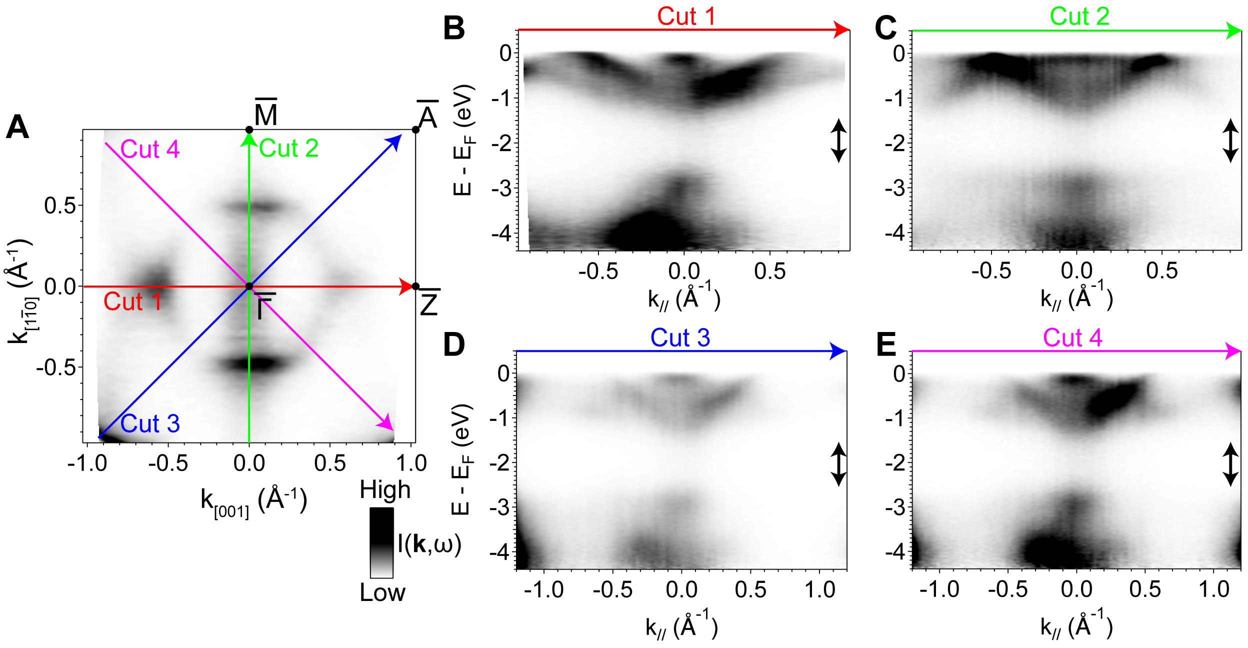

Figure S11: **Valence bands of the epitaxially-strained RuO_2 across a larger binding energy range.** (**A**) Measured Fermi surface similar to main text Fig. 4A with 62 eV *p*-polarized photons, but with all scans using a coarser energy step. Notice that the color scale for all plots here is enhanced by a same factor of 2 to emphasize regions with weak spectral intensity, as indicated by the color bar beneath panel (A). (**B**, **C**, **D**, **E**) Extracted electronic band dispersions along Cut 1, 2, 3, 4, respectively, as indicated by the colored arrows on panel (A). The vertical black double arrows in (B to E) indicate a region with suppressed density of states.

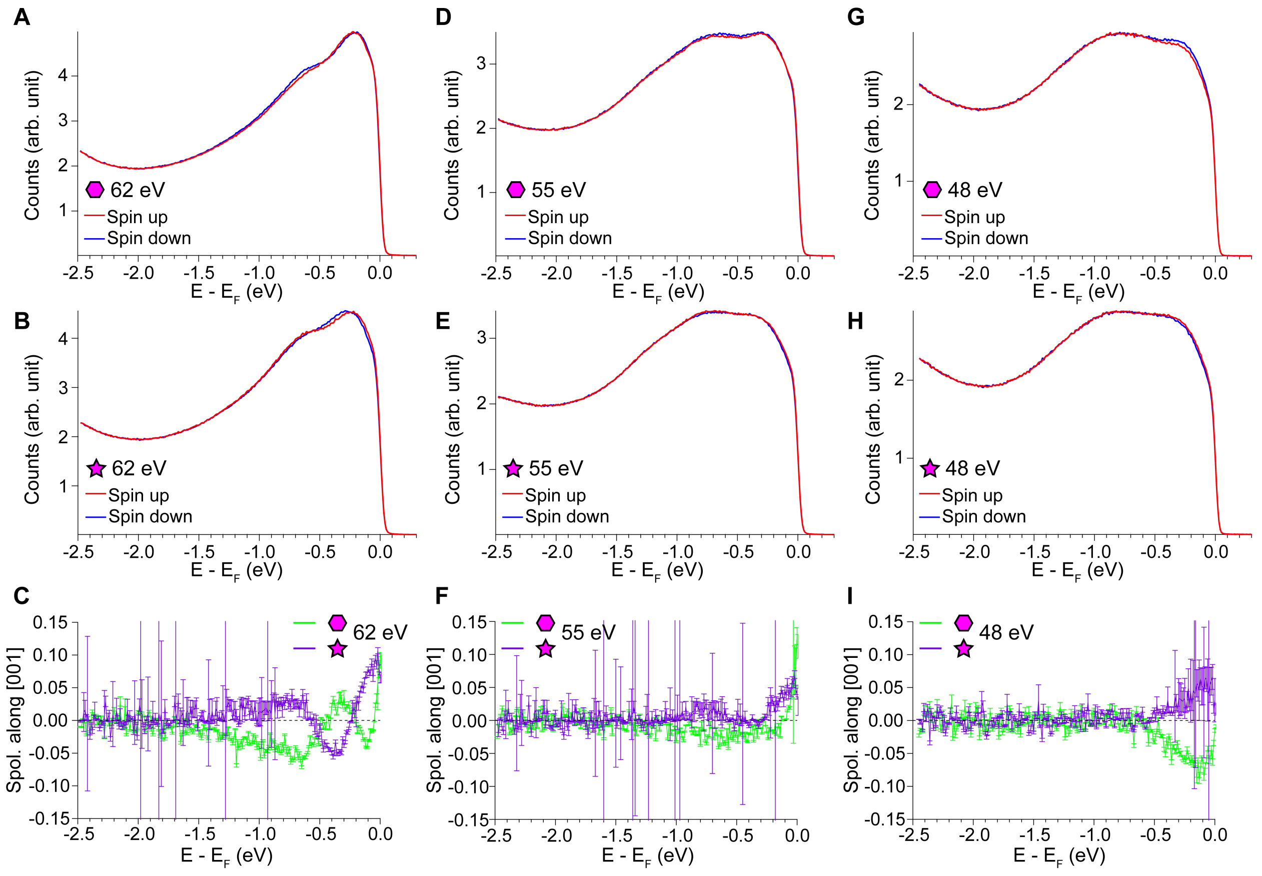

Figure S12: **Photon energy dependence for the finite-momenta spin-resolved feature in main text Fig. 4 (A to F).** (**A** and **B**) Spin-resolved energy distribution curves (EDCs) reproduced from Fig. 4 (C and D). (**C**) Photoelectron spin polarization along [001] reproduced from Fig. 4 (E and F) but now overlaid on a single plot. The photoemission angles chosen for panel (A to C) are from +7.5° and −7.5°. (**D**, **E**, **F**) Counterparts of (A to C) at 55 eV photon energy. Therefore, the photoelectron collection angle is moved to ±8.0° to keep the same in-plane momenta on the Fermi surface. (**G**, **H**, **I**) Counterparts of (A to C) at 48 eV photon energy, with the angle of photoelectron collection increased to ±8.8°.

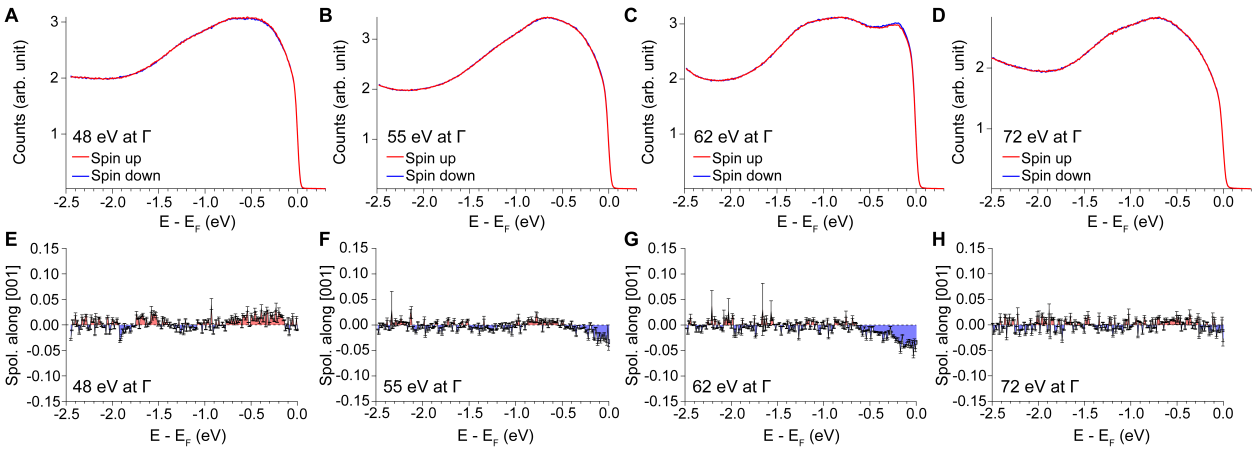

Figure S13: **Photon energy dependence for the normal emission spin-resolved feature discussed in main text Fig. 4 (G and H).** (**A**, **B**, **C**, **D**) Spin-resolved energy distribution curves measured at normal emission selecting photoelectrons polarized along the [001] direction at photon energies of 48, 55, 62, and 72 eV, respectively. (**E**, **F**, **G**, **H**) Calculated spin polarization $P_{[001]}$ at normal emission based on (A to D).

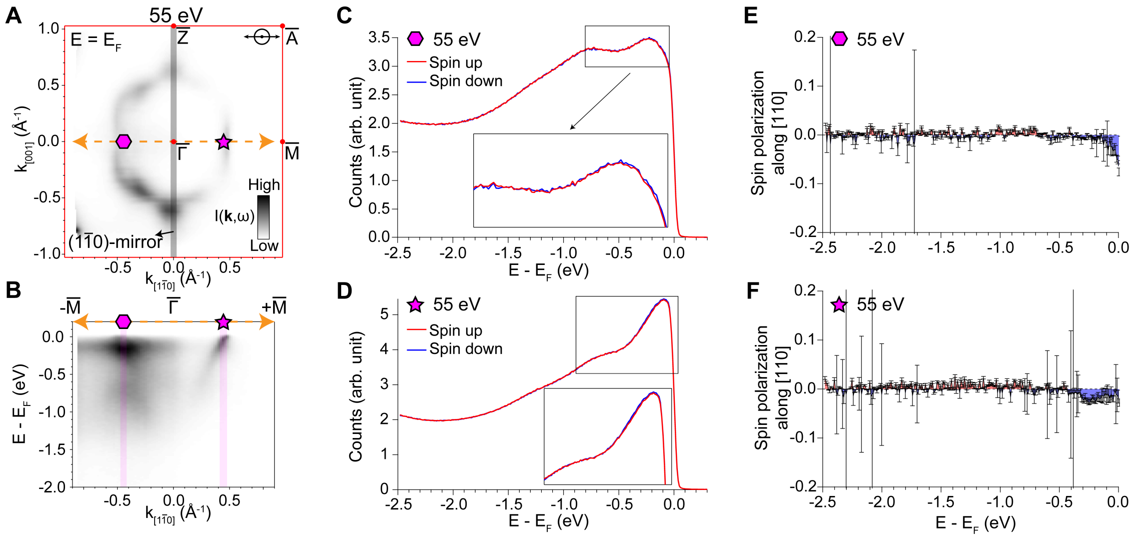

Figure S14: **Measured angle-resolved photoemission spectroscopy maps and photoelectron spin polarization along the out-of-plane [110] direction on the $\bar{\Gamma}-\bar{M}$ path at 55 eV with respect to the broken (1$\bar{1}$0)-mirror under Geometry B.** (**A**) Fermi surface probed by 55 eV photons. (**B**) Band dispersions along the $\bar{\Gamma}-\bar{M}$ direction indicated by the horizontal dashed double-arrow in (A). (**C** and **D**) Spin-resolved energy distribution curves integrated across the magenta bars on either sides of the (1$\bar{1}$0)-mirror in (B), selecting photoelectrons with spin polarization only along the out-of-plane [110] direction. (**E** and **F**) Converted spin polarization from (C and D), respectively.

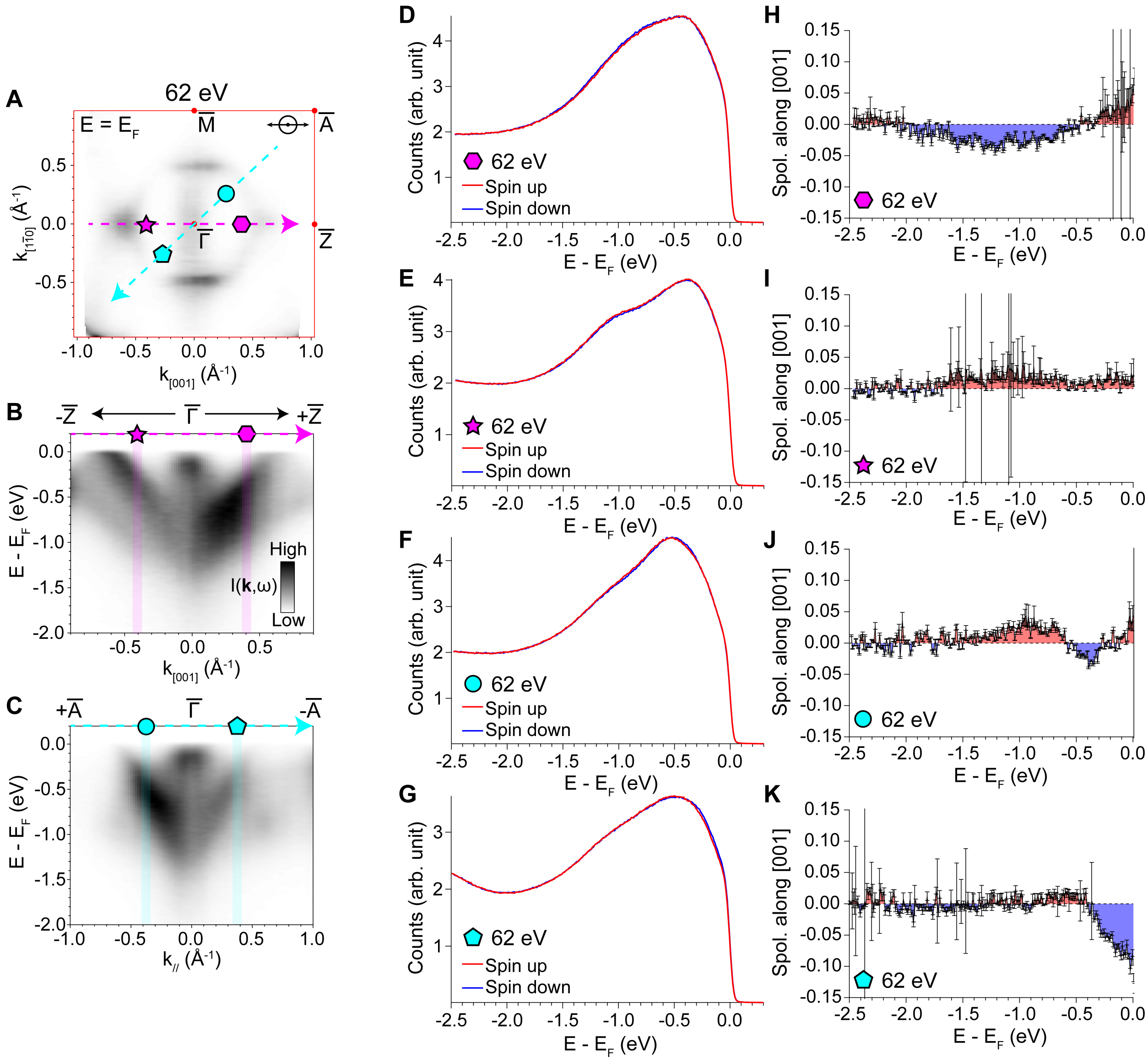

Figure S15: **Measured band dispersions and in-plane [001] photoelectron spin polarization on the $\bar{\Gamma} - \bar{Z}$ and $\bar{\Gamma} - \bar{A}$ paths using 62 eV light under Geometry A.** (**A**) Fermi surface reproduced from Fig. 4A. Magenta and cyan symbols indicate where the spin-resolved energy distribution curves (EDCs) are taken on $\bar{\Gamma} - \bar{Z}$ and $\bar{\Gamma} - \bar{A}$ lines. (**B** and **C**) Electronic band dispersions extracted along $\bar{\Gamma} - \bar{Z}$ and $\bar{\Gamma} - \bar{A}$, as indicated by the directional magenta and cyan arrows in panel (A). (**D**, **E**, **F**, **G**) Spin-resolved EDCs for the [001] photoelectrons spins measured at the four momenta indicated on (A). (**H**, **I**, **J**, **K**) Converted photoelectron spin polarization $P_{[001]}$ based on the data in (D to G).

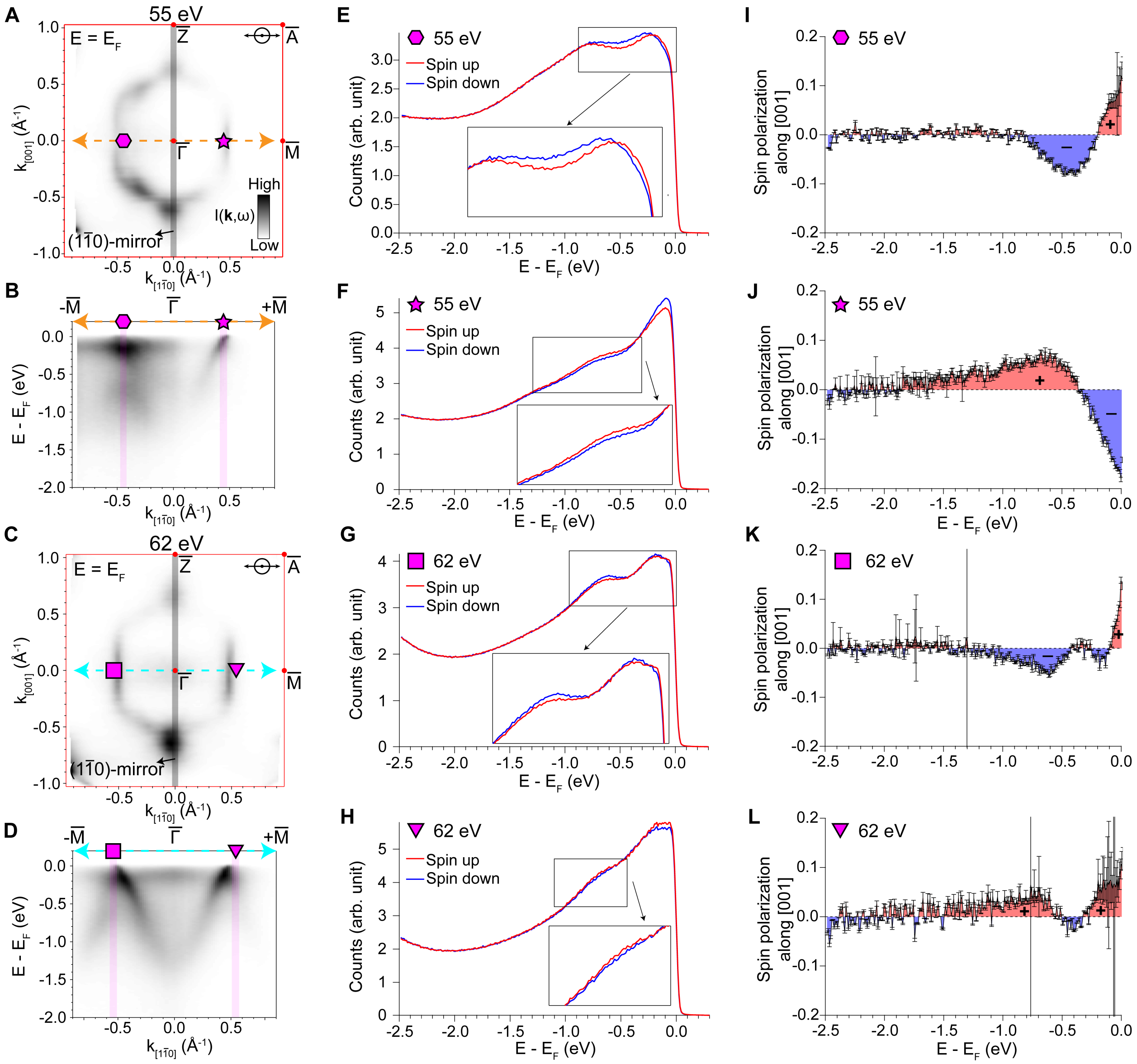

Figure S16: **Measured photoelectron spin polarization along the in-plane [001] direction on the $\bar{\Gamma} - \bar{M}$ path with respect to the broken (1$\bar{1}$0)-mirror plane under Geometry B.** (**A** and **B**) Fermi surface and band dispersions reproduced from Fig. S14 (A and B). (**C** and **D**) Same as (A and B), but taken using the 62 eV photon energy. The magenta symbols in (A to D) indicate where the spin-resolved energy distribution curves (EDCs) are measured. (**E** and **F**) Raw spin-resolved EDCs integrated across the magenta bars on either sides of the (1$\bar{1}$0)-mirror in panel (B), selecting photoelectrons with spin polarization only along the in-plane [001] direction. (**G** and **H**) Same as (E and F), but for 62 eV. (**I**, **J**, **K**, **L**) Converted spin polarization based on (E, F, G, H), respectively.

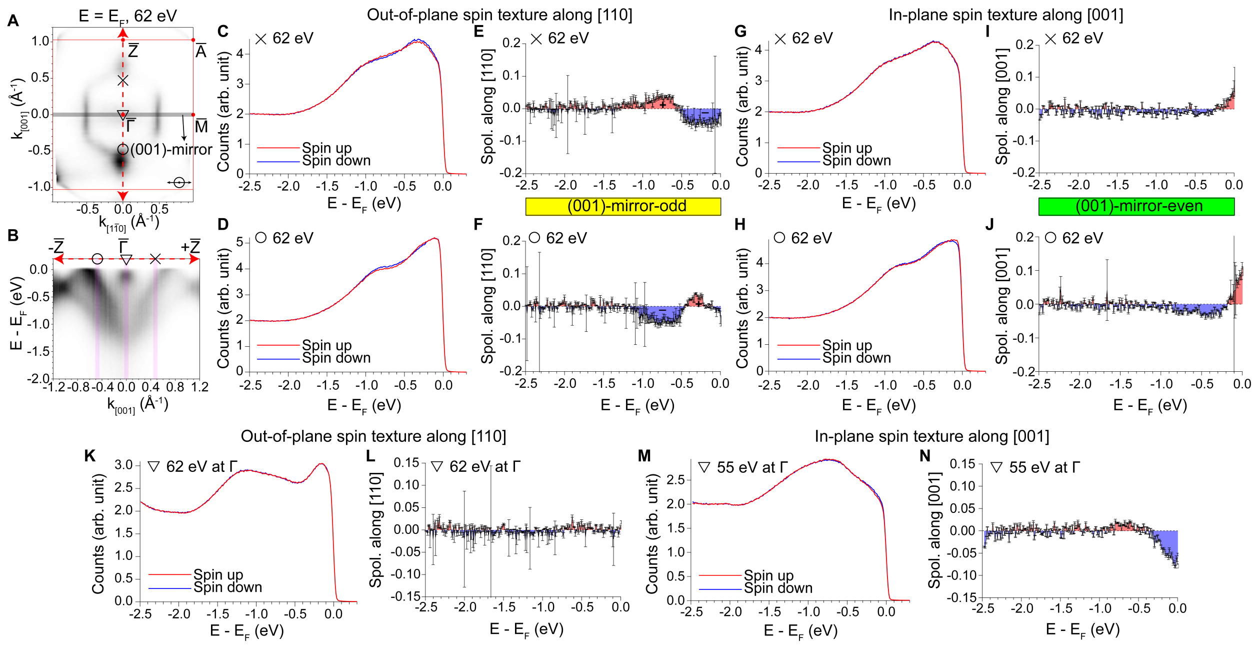

Figure S17: **Out-of-plane [110] and in-plane [001] photoelectron spin polarization on the $\bar{\Gamma} - \bar{Z}$ momentum path with respect to the preserved (001)-mirror plane measured under Geometry B.** (**A**) Fermi surface reproduced from Fig. S16C but highlighting the (001)-mirror and the momentum positions of the measured spin-resolved energy distribution curves (EDCs) using circular, cross, and triangular symbols. (**B**) Electronic band dispersions measured along $\bar{\Gamma} - \bar{Z}$ at 62 eV. The width of the vertical magenta bars provides an estimation of the momentum resolution in the spin-resolved measurement mode. (**C** and **D**) Spin-resolved EDCs selectively probing only the out-of-plane [110] spin polarization on the upper and lower sides of the (001)-mirror. (**E** and **F**) Converted out-of-plane spin polarization from (C and D), respectively. (**G**, **H**, **I**, **J**) Same as (C, D, E, F), but probing the in-plane spin polarization along [001]. (**K**) Spin-resolved EDCs selecting the out-of-plane [110] axis measured at normal emission using 62 eV light. (**L**) Converted [110] photoelectron spin polarization from (K). (**M** and **N**) Same as (K and L) but for the in-plane [001] spin quantization axis at normal emission using 55 eV photons.

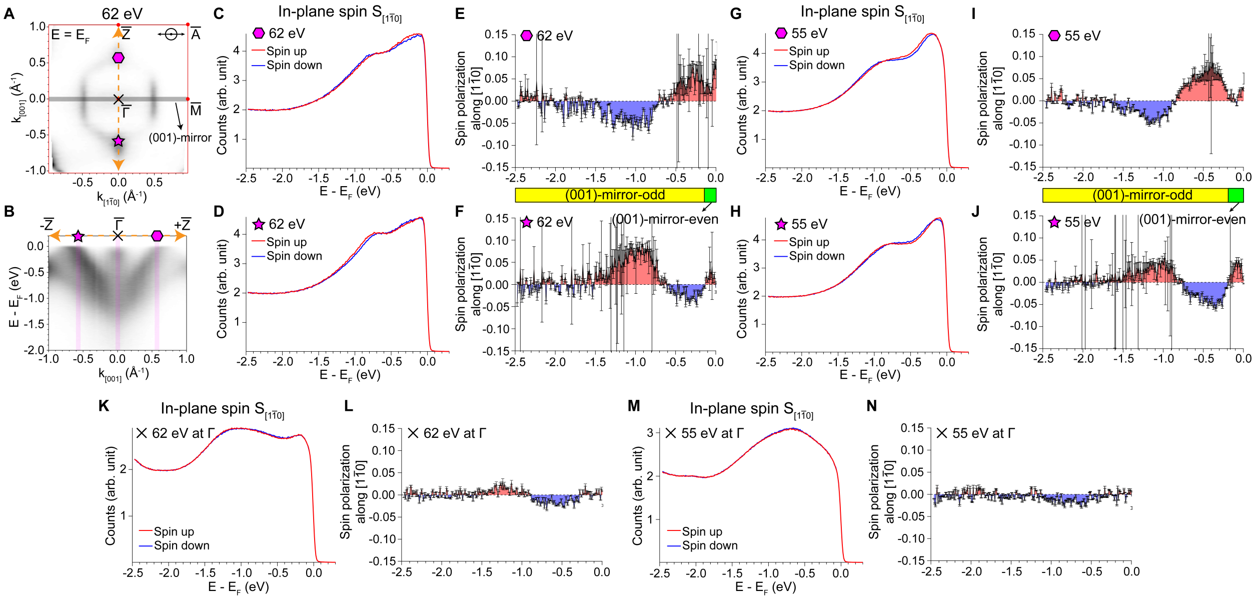

Figure S18: **Measured photoelectron spin polarization along the in-plane $[1\bar{1}0]$ axis on the $\bar{\Gamma} - \bar{Z}$ path with respect to the preserved (001)-mirror under Geometry B.** (**A**) Measured Fermi surface at 62 eV, which comes from the same sample measured in Fig. S15A but rotated azimuthally 90°. (**B**) Extracted $\bar{\Gamma} - \bar{Z}$ band structure. (**C** and **D**) $[1\bar{1}0]$ spin-resolved energy distribution curves (EDCs) measured at the momenta indicated by the pentagon and asterisk symbols respectively, mirror symmetric with respect to the preserved (001)-mirror. (**E** and **F**) Calculated $P_{[1\bar{1}0]}$ from (C and D). (**G** to **J**) Same as (C to F) but measured with 55 eV photons, while keeping the in-plane momenta near the Fermi level the same. (**K**) $[1\bar{1}0]$ spin-resolved EDCs at normal emission under 62 eV light. (**L**) $P_{[1\bar{1}0]}$ from data in (K). (**M** and **N**) Same as (K and L) but for normal emission $[1\bar{1}0]$ photoelectron spins under 55 eV photons.

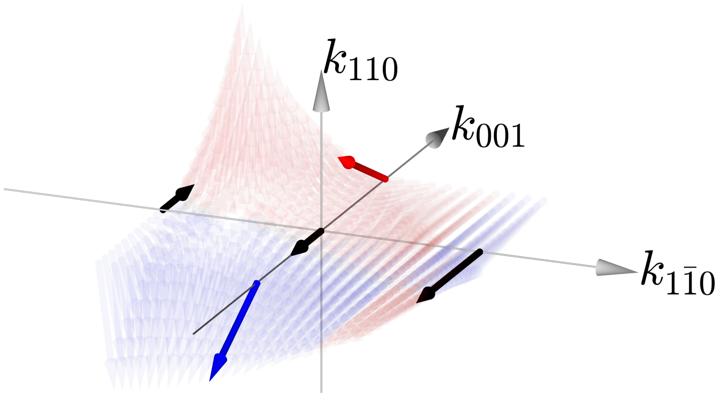

Figure S19: **Three-dimensional low-energy spin texture derived from the experiment-informed group theory analysis up to order 2 in k.**

$mm2.1'$	2_{110}	$m_{1\bar{1}0}$	m_{001}	σ_i	k_i	$k_i k_j$
$A_1^{\pm}$	1	1	1	·	k_{110}	$k_{1\bar{1}0}^2 - k_{001}^2,\ k_{110}^2$
$A_2^{\pm}$	1	-1	-1	σ_{110}	·	$k_{1\bar{1}0}\, k_{001}$
$B_1^{\pm}$	-1	-1	1	σ_{001}	$k_{1\bar{1}0}$	$k_{110}\, k_{1\bar{1}0}$
$B_2^{\pm}$	-1	1	-1	$\sigma_{1\bar{1}0}$	k_{001}	$k_{110}\, k_{001}$

Table S1: **Character table for the magnetic point group $mm2.1'$ the point group of strained, polar RuO_2.** Columns indicate symmetry eigenvalues under each generator, and allowed linear/quadratic k- and σ-terms. The superscript ± denotes whether the irrep is even/odd under time reversal $1'$. Note that the Geometry A used in our experiments breaks the m_{001} mirror, and as such generates terms that transform as B_2^+. Similarly, Geometry B breaks the $m_{1\bar{1}0}$ mirror, generating terms that transform as B_1^+.

File S1 DFT (PBE) relaxed crystal structure of the 15-layer inversion-symmetric slab of RuO_2.

File S2 DFT (PBE) relaxed crystal structure of the 29-layer inversion symmetric slab of the RuO_2/TiO_2 heterostructure.

REFERENCES

1. L. Šmejkal, J. Sinova, T. Jungwirth, Beyond conventional ferromagnetism and antiferromagnetism: A phase with nonrelativistic spin and crystal rotation symmetry. *Phys. Rev. X* **12**, 031042 (2022).

2. L. Šmejkal, J. Sinova, T. Jungwirth, Emerging research landscape of altermagnetism. *Phys. Rev. X* **12**, 040501 (2022).

3. T. Jungwirth, R. M. Fernandes, J. Sinova, L. Smejkal, Altermagnets and beyond: Nodal magnetically-ordered phases. arXiv:2409.10034 [cond-mat.mtrl-sci] (2024).

4. C. Wu, S.-C. Zhang, Dynamic generation of spin-orbit coupling. *Phys. Rev. Lett.* **93**, 036403 (2004).

5. C. Wu, K. Sun, E. Fradkin, S.-C. Zhang, Fermi liquid instabilities in the spin channel. *Phys. Rev. B* **75**, 115103 (2007).

6. Z. Xiao, J. Zhao, Y. Li, R. Shindou, Z.-D. Song, Spin space groups: Full classification and applications. *Phys. Rev. X* **14**, 031037 (2024).

7. X. Chen, J. Ren, Y. Zhu, Y. Yu, A. Zhang, P. Liu, J. Li, Y. Liu, C. Li, Q. Liu, Enumeration and representation theory of spin space groups. *Phys. Rev. X* **14**, 031038 (2024).

8. Y. Jiang, Z. Song, T. Zhu, Z. Fang, H. Weng, Z.-X. Liu, J. Yang, C. Fang, Enumeration of spin-space groups: Toward a complete description of symmetries of magnetic orders. *Phys. Rev. X* **14**, 031039 (2024).

9. A. B. Hellenes, T. Jungwirth, R. Jaeschke-Ubiergo, A. Chakraborty, J. Sinova, L. Šmejkal, P-wave magnets. arXiv:2309.01607 [cond-mat.mes-hall] (2023).

10. P. Liu, J. Li, J. Han, X. Wan, Q. Liu, Spin-group symmetry in magnetic materials with negligible spin-orbit coupling. *Phys. Rev. X* **12**, 021016 (2022).

11. D. S. Antonenko, R. M. Fernandes, J. W. F. Venderbos, Mirror chern bands and weyl nodal loops in altermagnets. *Phys. Rev. Lett.* **134**, 096703 (2025).

12. X.-J. Gao, Z.-T. Sun, R.-P. Yu, X.-Y. Guo, K. T. Law, Heesch Weyl fermions in inadmissible chiral antiferromagnets. arXiv:2305.15876 [cond-mat.mtrl-sci] (2023).

13. R. M. Fernandes, V. S. de Carvalho, T. Birol, R. G. Pereira, Topological transition from nodal to nodeless Zeeman splitting in altermagnets. *Phys. Rev. B* **109**, 024404 (2024).

14. M. Hu, O. Janson, C. Felser, P. McClarty, J. van den Brink, M. G. Vergniory, Spin Hall and Edelstein effects in chiral non-collinear altermagnets. *Nat. Commun.* **16**, 8529 (2025).

15. P. G. Radaelli, G. Gurung, Color symmetry and altermagneticlike spin textures in noncollinear antiferromagnets. *Phys. Rev. B* **112**, 014431 (2025).

16. L. Šmejkal, A. Marmodoro, K.-H. Ahn, R. González-Hernández, I. Turek, S. Mankovsky, H. Ebert, S. W. D'Souza, O. Šipr, J. Sinova, T. Jungwirth, Chiral magnons in altermagnetic RuO_2. *Phys. Rev. Lett.* **131**, 256703 (2023).

17. Z. Liu, M. Ozeki, S. Asai, S. Itoh, T. Masuda, Chiral split magnon in altermagnetic MnTe. *Phys. Rev. Lett.* **133**, 156702 (2024).

18. Y.-F. Zhang, X.-S. Ni, K. Chen, K. Cao, Chiral magnon splitting in altermagnetic CrSb from first principles. *Phys. Rev. B* **111**, 174451 (2025).

19. L. Šmejkal, R. González-Hernández, T. Jungwirth, J. Sinova, Crystal time-reversal symmetry breaking and spontaneous Hall effect in collinear antiferromagnets. *Sci. Adv.* **6**, eaaz8809 (2020).

20. T. Sato, S. Haddad, I. C. Fulga, F. F. Assaad, J. van den Brink, Altermagnetic anomalous Hall effect emerging from electronic correlations. *Phys. Rev. Lett.* **133**, 086503 (2024).

21. K. Takahashi, C. R. W. Steward, M. Ogata, R. M. Fernandes, J. Schmalian, Elasto-Hall conductivity and the anomalous Hall effect in altermagnets. *Phys. Rev. B* **111**, 184408 (2025).

22. R. González-Hernández, P. Ritzinger, K. Výborný, J. Železný, A. Manchon, Non-relativistic torque and Edelstein effect in non-collinear magnets. *Nat. Commun.* **15**, 7663 (2024).

23. L. Šmejkal, A. B. Hellenes, R. González-Hernández, J. Sinova, T. Jungwirth, Giant and tunneling magnetoresistance in unconventional collinear antiferromagnets with nonrelativistic spin-momentum coupling. *Phys. Rev. X* **12**, 011028 (2022).

24. A. Dal Din, O. J. Amin, P. Wadley, K. W. Edmonds, Antiferromagnetic spintronics and beyond. *Npj Spintron.* **2**, 25 (2024).

25. Q. Liu, X. Dai, S. Blügel, Different facets of unconventional magnetism. *Nat. Phys.* **21**, 329–331 (2025).

26. M. Weißenhofer, A. Marmodoro, Atomistic spin dynamics simulations of magnonic spin Seebeck and spin Nernst effects in altermagnets. *Phys. Rev. B* **110**, 094427 (2024).

27. T. Jungwirth, J. Sinova, P. Wadley, D. Kriegner, H. Reichlova, F. Krizek, H. Ohno, L. Šmejkal, Altermagnetic spintronics. arXiv:2508.09748 [cond-mat.mtrl-sci] (2025).

28. L. Šmejkal, Altermagnetic multiferroics and altermagnetoelectric effect. arXiv:2411.19928 [cond-mat.mtrl-sci] (2024).

29. Z. H. Zhu, J. Strempfer, R. R. Rao, C. A. Occhialini, J. Pelliciari, Y. Choi, T. Kawaguchi, H. You, J. F. Mitchell, Y. Shao-Horn, R. Comin, Anomalous antiferromagnetism in metallic RuO_2 determined by resonant X-ray scattering. *Phys. Rev. Lett.* **122**, 017202 (2019).

30. T. Berlijn, P. C. Snijders, O. Delaire, H. D. Zhou, T. A. Maier, H.-B. Cao, S.-X. Chi, M. Matsuda, Y. Wang, M. R. Koehler, P. R. C. Kent, H. H. Weitering, Itinerant antiferromagnetism in RuO_2. *Phys. Rev. Lett.* **118**, 077201 (2017).

31. K.-H. Ahn, A. Hariki, K.-W. Lee, J. Kuneš, Antiferromagnetism in RuO_2 as *d*-wave Pomeranchuk instability. *Phys. Rev. B* **99**, 184432 (2019).

32. Y. Guo, H. Liu, O. Janson, I. C. Fulga, J. van den Brink, J. I. Facio, Spin-split collinear antiferromagnets: A large-scale ab-initio study. *Mater. Today Phys.* **32**, 100991 (2023).

33. Z. Feng, X. Zhou, L. Šmejkal, L. Wu, Z. Zhu, H. Guo, R. González-Hernández, X. Wang, H. Yan, P. Qin, X. Zhang, H. Wu, H. Chen, Z. Meng, L. Liu, Z. Xia, J. Sinova, T. Jungwirth, Z.

Liu, An anomalous Hall effect in altermagnetic ruthenium dioxide. *Nat. Electron.* **5**, 735–743 (2022).

34. T. Tschirner, P. Keßler, R. D. Gonzalez Betancourt, T. Kotte, D. Kriegner, B. Büchner, J. Dufouleur, M. Kamp, V. Jovic, L. Smejkal, J. Sinova, R. Claessen, T. Jungwirth, S. Moser, H. Reichlova, L. Veyrat, Saturation of the anomalous Hall effect at high magnetic fields in altermagnetic RuO_2. *APL Mater.* **11**, 101103 (2023).

35. J. Song, S. H. Lee, S. Kang, D. Kim, J. H. Jeong, T. Oh, S. Lee, S. Lee, S. Lee, K.-H. Ahn, K.-W. Lee, M. Kim, T. W. Noh, B.-J. Yang, C. Kim, Spin-orbit coupling driven magnetic response in altermagnetic RuO_2. *Small* **21**, e2407722 (2025).

36. H. Chen, Z.-A. Wang, P. Qin, Z. Meng, X. Zhou, X. Wang, L. Liu, G. Zhao, Z. Duan, T. Zhang, J. Liu, D.-F. Shao, C. Jiang, Z. Liu, Spin-splitting magnetoresistance in altermagnetic RuO_2 thin films. *Adv. Mater.* **37**, e2507764 (2025).

37. H. Bai, L. Han, X. Y. Feng, Y. J. Zhou, R. X. Su, Q. Wang, L. Y. Liao, W. X. Zhu, X. Z. Chen, F. Pan, X. L. Fan, C. Song, Observation of spin splitting torque in a collinear antiferromagnet RuO_2. *Phys. Rev. Lett.* **128**, 197202 (2022).

38. S. Karube, T. Tanaka, D. Sugawara, N. Kadoguchi, M. Kohda, J. Nitta, Observation of spin-splitter torque in collinear antiferromagnetic RuO_2. *Phys. Rev. Lett.* **129**, 137201 (2022).

39. A. Bose, N. J. Schreiber, R. Jain, D.-F. Shao, H. P. Nair, J. Sun, X. S. Zhang, D. A. Muller, E. Y. Tsymbal, D. G. Schlom, D. C. Ralph, Tilted spin current generated by the collinear antiferromagnet ruthenium dioxide. *Nat. Electron.* **5**, 267–274 (2022).

40. Y. Zhang, H. Bai, L. Han, C. Chen, Y. Zhou, C. H. Back, F. Pan, Y. Wang, C. Song, Simultaneous high charge-spin conversion efficiency and large spin diffusion length in altermagnetic RuO_2. *Adv. Funct. Mater.* **34**, 2313332 (2024).

41. X. Feng, H. Bai, X. Fan, M. Guo, Z. Zhang, G. Chai, T. Wang, D. Xue, C. Song, X. Fan, Incommensurate spin density wave in antiferromagnetic RuO_2 evinced by abnormal spin splitting torque. *Phys. Rev. Lett.* **132**, 086701 (2024).

42. H. Bai, Y. C. Zhang, Y. J. Zhou, P. Chen, C. H. Wan, L. Han, W. X. Zhu, S. X. Liang, Y. C. Su, X. F. Han, F. Pan, C. Song, Efficient spin-to-charge conversion via altermagnetic spin splitting effect in antiferromagnet RuO_2. *Phys. Rev. Lett.* **130**, 216701 (2023).

43. C.-T. Liao, Y.-C. Wang, Y.-C. Tien, S.-Y. Huang, D. Qu, Separation of inverse altermagnetic spin-splitting effect from inverse spin Hall effect in RuO_2. *Phys. Rev. Lett.* **133**, 056701 (2024).

44. O. Fedchenko, J. Minár, A. Akashdeep, S. W. D'Souza, D. Vasilyev, O. Tkach, L. Odenbreit, Q. Nguyen, D. Kutnyakhov, N. Wind, L. Wenthaus, M. Scholz, K. Rossnagel, M. Hoesch, M. Aeschlimann, B. Stadtmüller, M. Kläui, G. Schönhense, T. Jungwirth, A. B. Hellenes, G. Jakob, L. Šmejkal, J. Sinova, H.-J. Elmers, Observation of time-reversal symmetry breaking in the band structure of altermagnetic RuO_2. *Sci. Adv.* **10**, eadj4883 (2024).

45. Y. Lytvynenko, A. Akashdeep, T. P. Vo, O. Tkach, S. V. Chernov, A. Gloskovskii, C. Schlueter, C. Luo, V. Ukleev, F. Radu, F. Kronast, T. Hiroto, A. Winkelmann, J. Minár, M. Kläui, G. Schönhense, G. Jakob, H. J. Elmers, O. Fedchenko, Magnetic circular dichroism in core-level x-ray photoelectron spectroscopy of altermagnetic RuO_2 films. *Phys. Rev. B* **113**, 014403 (2026).

46. Z. Lin, D. Chen, W. Lu, X. Liang, S. Feng, K. Yamagami, J. Osiecki, M. Leandersson, B. Thiagarajan, J. Liu, C. Felser, J. Ma, Observation of giant spin splitting and d-wave spin texture in room temperature altermagnet RuO_2. arXiv:2402.04995 [cond-mat.mtrl-sci] (2024).

47. P. Keßler, L. Garcia-Gassull, A. Suter, T. Prokscha, Z. Salman, D. Khalyavin, P. Manuel, F. Orlandi, I. I. Mazin, R. Valentí, S. Moser, Absence of magnetic order in RuO_2: Insights from μSR spectroscopy and neutron diffraction. *Npj Spintron.* **2**, 50 (2024).

48. M. Hiraishi, H. Okabe, A. Koda, R. Kadono, T. Muroi, D. Hirai, Z. Hiroi, Nonmagnetic ground state in RuO_2 revealed by muon spin rotation. *Phys. Rev. Lett.* **132**, 166702 (2024).

49. L. Kiefer, F. Wirth, A. Bertin, P. Becker, L. Bohatý, K. Schmalzl, A. Stunault, J. A. Rodríguez-Velamazan, O. Fabelo, M. Braden, Crystal structure and absence of magnetic order in single-crystalline RuO_2. *J. Phys. Condens. Matter* **37**, 135801 (2025).

50. M. Wenzel, E. Uykur, S. Rößler, M. Schmidt, O. Janson, A. Tiwari, M. Dressel, A. A. Tsirlin, Fermi-liquid behavior of nonaltermagnetic RuO_2. *Phys. Rev. B* **111**, L041115 (2025).

51. Z. Wu, M. Long, H. Chen, S. Paul, H. Matsuki, O. Zheliuk, U. Zeitler, G. Li, R. Zhou, Z. Zhu, D. Graf, T. I. Weinberger, F. M. Grosche, Y. Maeno, A. G. Eaton, Fermi surface of RuO_2 measured by quantum oscillations. *Phys. Rev. X* **15**, 031044 (2025).

52. T. Qian, A. Rutherford, E. S. Choi, H. Zhou, B. Maiorov, M. Lee, C. A. Mizzi, Determining the nature of magnetism in altermagnetic candidate RuO_2. arXiv:2504.21138 [cond-mat.mtrl-sci] (2025).

53. G. Yumnam, P. R. Raghuvanshi, J. D. Budai, L. Bocklage, D. Abernathy, Y. Cheng, A. H. Said, I. I. Mazin, H. Zhou, B. A. Frandsen, D. S. Parker, L. R. Lindsay, V. R. Cooper, M. E. Manley, R. P. Hermann, Constraints on magnetism and correlations in RuO_2 from lattice dynamics and Mössbauer spectroscopy. *Cell Rep. Phys. Sci.* **6**, 102852 (2025).

54. Y. C. Wang, Z. Y. Shen, C. H. Lin, W. C. Hsu, Y. S. Chen, Y. Y. Chin, A. K. Singh, W. L. Lee, C. T. Chen, S. Y. Huang, D. Qu, Absence of transport altermagnetic spin-splitting effect in RuO_2. *Nano Lett.* **26**, 2548–2554 (2026).

55. D. T. Plouff, L. Scheuer, S. Shrestha, W. Wu, N. J. Parvez, S. Bhatt, X. Wang, L. Gundlach, M. B. Jungfleisch, J. Q. Xiao, Revisiting altermagnetism in RuO_2: A study of laser-pulse induced charge dynamics by time-domain terahertz spectroscopy. *Npj Spintron.* **3**, 17 (2025).

56. J. Liu, J. Zhan, T. Li, J. Liu, S. Cheng, Y. Shi, L. Deng, M. Zhang, C. Li, J. Ding, Q. Jiang, M. Ye, Z. Liu, Z. Jiang, S. Wang, Q. Li, Y. Xie, Y. Wang, S. Qiao, J. Wen, Y. Sun, D. Shen, Absence of altermagnetic spin splitting character in rutile oxide RuO_2. *Phys. Rev. Lett.* **133**, 176401 (2024).

57. T. Osumi, K. Yamauchi, S. Souma, S. Paul, A. Honma, K. Nakayama, K. Ozawa, M. Kitamura, K. Horiba, H. Kumigashira, C. Bigi, F. Bertran, T. Oguchi, T. Takahashi, Y. Maeno, T. Sato, Spin-degenerate bulk bands and topological surface states associated with Dirac nodal lines in RuO_2. *Phys. Rev. B* **113**, 085116 (2026).

58. A. Smolyanyuk, I. I. Mazin, L. Garcia-Gassull, R. Valentí, Fragility of the magnetic order in the prototypical altermagnet RuO_2. *Phys. Rev. B* **109**, 134424 (2024).

59. Z. Qian, Y. Yang, S. Liu, C. Wu, Fragile unconventional magnetism in RuO_2 by proximity to Landau-Pomeranchuk instability. *Phys. Rev. B* **111**, 174425 (2025).

60. S. G. Jeong, I. H. Choi, S. Nair, L. Buiarelli, B. Pourbahari, J. Y. Oh, B. Y. X. Lin, J. M. Le Beau, N. Bassim, D. Hirai, A. Seo, W. S. Choi, R. M. Fernandes, T. Birol, L. Zhao, J. S. Lee, B. Jalan, Altermagnetic polar metallic phase in ultrathin epitaxially strained RuO_2 films. *Proc. Natl. Acad. Sci. U.S.A.* **123**, e2526641123 (2026).

61. A. K. Rajapitamahuni, S. Nair, Z. Yang, A. K. Manjeshwar, S. G. Jeong, W. Nunn, B. Jalan, Thickness-dependent insulator-to-metal transition in epitaxial RuO_2 films. *Phys. Rev. Mater.* **8**, 075002 (2024).

62. M. Weber, S. Wust, L. Haag, P. Herrgen, A. Akashdeep, K. Leckron, C. Schmitt, R. Ramos, T. Kikkawa, E. Saitoh, M. Kläui, L. Šmejkal, J. Sinova, M. Aeschlimann, G. Jakob, B. Stadtmüller, H. C. Schneider, All optical excitation of spin polarization in d-wave altermagnets. arXiv:2408.05187 [cond-mat.mtrl-sci] (2024).

63. S. G. Jeong, S. Lee, B. Lin, Z. Yang, I. H. Choi, J. Y. Oh, S. Song, S. W. Lee, S. Nair, R. Choudhary, J. Parikh, S. Park, W. S. Choi, J. S. Lee, J. M. Le Beau, T. Low, B. Jalan, Metallicity and anomalous Hall effect in epitaxially strained, atomically thin RuO_2 films. *Proc. Natl. Acad. Sci. U.S.A.* **122**, e2500831122 (2025).

64. M. Meinert, A meta-GGA perpective on the altermagnetism of RuO_2. arXiv:2512.04995 [cond-mat.mtrl-sci] (2025).

65. F. Yang, R. M. Fernandes, T. Birol, Symmetries of spin-splitting induced by spin-orbit coupling in non-magnetic crystals. arXiv:2602.12396 [cond-mat.mtrl-sci] (2026).

66. E. Tamura, W. Piepke, R. Feder, New spin-polarization effect in photoemission from nonmagnetic surfaces. *Phys. Rev. Lett.* **59**, 934–937 (1987).

67. E. Tamura, R. Feder, Spin polarization in normal photoemission by linearly polarized light from nonmagnetic (001) surfaces. *Europhys. Lett.* **16**, 695–700 (1991).

68. J. Henk, R. Feder, Spin polarization in normal photoemission by linearly polarized light from non-magnetic (110) surfaces. *Europhys. Lett.* **28**, 609–614 (1994).

69. B. Schmiedeskamp, B. Vogt, U. Heinzmann, Experimental verification of a new spin-polarization effect in photoemission: Polarized photoelectrons from Pt(111) with linearly polarized radiation in normal incidence and normal emission. *Phys. Rev. Lett.* **60**, 651–654 (1988).

70. N. Irmer, R. David, B. Schmiedeskamp, U. Heinzmann, Experimental verification of a spin effect in photoemission: Polarized electrons due to phase-shift differences in the normal emission from Pt(100) by unpolarized radiation. *Phys. Rev. B* **45**, 3849–3852 (1992).

71. N. Irmer, F. Frentzen, S.-W. Yu, B. Schmiedeskamp, U. Heinzmann, A new effect in spin-resolved photoemission from Pt(110) in normal emission by linearly polarized VUV-radiation. *J. Electron Spectrosc. Relat. Phenom.* **78**, 321–324 (1996).

72. R. Feder, J. Kirschner, Spin polarization in directional photoemission from non-magnetic crystals by unpolarized light. *Solid State Commun.* **40**, 547–550 (1981).

73. U. Heinzmann, J. H. Dil, Spin-orbit-induced photoelectron spin polarization in angle-resolved photoemission from both atomic and condensed matter targets. *J. Phys. Condens. Matter* **24**, 173001 (2012).

74. W. Nunn, S. Nair, H. Yun, A. Kamath Manjeshwar, A. Rajapitamahuni, D. Lee, K. A. Mkhoyan, B. Jalan, Solid-source metal-organic molecular beam epitaxy of epitaxial RuO_2. *APL Mater.* **9**, 091112 (2021).

75. V. Jovic, R. J. Koch, S. K. Panda, H. Berger, P. Bugnon, A. Magrez, K. E. Smith, S. Biermann, C. Jozwiak, A. Bostwick, E. Rotenberg, S. Moser, Dirac nodal lines and flat-band surface state in the functional oxide RuO_2. *Phys. Rev. B* **98**, 241101 (2018).

76. J. P. Ruf, H. Paik, N. J. Schreiber, H. P. Nair, L. Miao, J. K. Kawasaki, J. N. Nelson, B. D. Faeth, Y. Lee, B. H. Goodge, B. Pamuk, C. J. Fennie, L. F. Kourkoutis, D. G. Schlom, K. M. Shen, Strain-stabilized superconductivity. *Nat. Commun.* **12**, 59 (2021).

77. V. Jovic, A. Consiglio, K. E. Smith, C. Jozwiak, A. Bostwick, E. Rotenberg, D. di Sante, S. Moser, Momentum for catalysis: How surface reactions shape the RuO_2 flat surface state. *ACS Catal.* **11**, 1749–1757 (2021).

78. P. Keßler, A. Feuerpfeil, A. Consiglio, H. Hohmann, R. Thomale, J. Erhardt, B. Liu, V. Jovic, R. Claessen, P. Härtl, M. Dürrnagel, S. Moser, Moiré-assisted charge instability in ultrathin RuO_2. arXiv:2507.05047 [cond-mat.mtrl-sci] (2025).

79. A. Akashdeep, S. Krishnia, J. H. Ha, S. An, M. Gaerner, T. Prokscha, A. Suter, G. Janka, G. Reiss, T. Kuschel, D. S. Han, A. di Bernardo, Z. Salman, G. Jakob, M. Kläui, Surface-localized magnetic order in RuO_2 thin films revealed by low-energy muon probes. *Appl. Phys. Lett.* **128**, 022406 (2026).

80. S. G. Jeong, S. Lee, J. Y. Oh, B. Y. X. Lin, A. Santhosh, J. M. Le Beau, A. J. Grutter, W. S. Choi, T. Low, V. Lauter, B. Jalan, Emergence of unconventional magnetic order in strain-engineered RuO_2/TiO_2 superlattices. arXiv:2601.10518 [cond-mat.mtrl-sci] (2026).

81. Y.-M. Xie, X.-J. Gao, X. Y. Xu, C.-P. Zhang, J.-X. Hu, J. Z. Gao, K. T. Law, Kramers nodal line metals. *Nat. Commun.* **12**, 3064 (2021).

82. Y. Zhang, Y. Gao, X.-J. Gao, S. Lei, Z. Ni, J. S. Oh, J. Huang, Z. Yue, M. Zonno, S. Gorovikov, M. Hashimoto, D. Lu, J. D. Denlinger, R. J. Birgeneau, J. Kono, L. Wu, K. T. Law, E. Morosan, M. Yi, Kramers nodal lines and Weyl fermions in SmAlSi. *Commun. Phys.* **6**, 134 (2023).

83. Y. Zhang, Y. Gao, A. Pulkkinen, X. Guo, J. Huang, Y. Guo, Z. Yue, J. S. Oh, A. Moon, M. Oudah, X.-J. Gao, A. Marmodoro, A. Fedorov, S.-K. Mo, M. Hashimoto, D. Lu, A. Rajapitamahuni, E. Vescovo, J. Kono, A. M. Hallas, R. J. Birgeneau, L. Balicas, J. Minár, P. Hosur, K. T. Law, E. Morosan, M. Yi, Kramers nodal lines in intercalated TaS_2 superconductors. *Nat. Commun.* **16**, 4984 (2025).

84. G. Domaine, M. M. Hirschmann, K. Parshukov, M. Date, H. L. Meyerheim, M. D. Watson, K. Mohseni, S. K. Y. Dufresne, S. Terakawa, M. Rosmus, N. Olszowska, S. S. P. Parkin, A. P. Schnyder, N. B. M. Schröter, Tunable Octdong and Spindle-torus Fermi surfaces in Kramers nodal line metals. *Nat. Commun.* **16**, 11128 (2025).

85. I. Waluyo, A. Hunt, Ambient pressure X-ray photoelectron spectroscopy at the IOS (23-ID-2) beamline at the national synchrotron light source II. *Synchrotron Radiat. News* **35**, 31–38 (2022).

86. W. Kohn, L. J. Sham, Self-consistent equations including exchange and correlation effects. *Phys. Rev.* **140**, A1133–A1138 (1965).

87. G. Kresse, J. Furthmüller, Efficient iterative schemes for ab initio total-energy calculations using a plane-wave basis set. *Phys. Rev. B* **54**, 11169–11186 (1996).

88. P. E. Blöchl, Projector augmented-wave method. *Phys. Rev. B* **50**, 17953–17979 (1994).

89. G. Kresse, D. Joubert, From ultrasoft pseudopotentials to the projector augmented-wave method. *Phys. Rev. B* **59**, 1758–1775 (1999).

90. J. P. Perdew, K. Burke, M. Ernzerhof, Generalized gradient approximation made simple. *Phys. Rev. Lett.* **77**, 3865–3868 (1996).

91. J. K. Kawasaki, C. H. Kim, J. N. Nelson, S. Crisp, C. J. Zollner, E. Biegenwald, J. T. Heron, C. J. Fennie, D. G. Schlom, K. M. Shen, Engineering carrier effective masses in ultrathin quantum wells of IrO_2. *Phys. Rev. Lett.* **121**, 176802 (2018).

92. P. Blaha, K. Schwarz, F. Tran, R. Laskowski, G. K. H. Madsen, L. D. Marks, WIEN2k: An APW+lo program for calculating the properties of solids. *J. Chem. Phys.* **152**, 074101 (2020).

93. H. Ebert, D. Ködderitzsch, J. Minár, Calculating condensed matter properties using the KKR-Green's function method—Recent developments and applications. *Rep. Prog. Phys.* **74**, 096501 (2011).

94. J. Braun, J. Minár, H. Ebert, Correlation, temperature and disorder: Recent developments in the one-step description of angle-resolved photoemission. *Phys. Rep.* **740**, 1–34 (2018).

95. J. Rundgren, G. Malmstrom, Transmission and reflection of low-energy electrons at the surface barrier of a metal. *J. Phys. C Solid State Phys.* **10**, 4671–4687 (1977).

96. Y. Kaga, Y. Abe, H. Yanagisawa, M. Kawamura, K. Sasaki, Ru and RuO_2 Thin films by XPS. *Surf. Sci. Spectra* **6**, 68–74 (1999).

97. Y. Kaga, Y. Abe, M. Kawamura, K. Sasaki, Thermal stability of RuO thin films and effects of annealing ambient on their reduction process. *Jpn. J. Appl. Phys.* **38**, 3689–3692 (1999).

98. H. Over, A. P. Seitsonen, E. Lundgren, M. Wiklund, J. N. Andersen, Spectroscopic characterization of catalytically active surface sites of a metallic oxide. *Chem. Phys. Lett.* **342**, 467–472 (2001).

99. H. Over, A. P. Seitsonen, E. Lundgren, M. Smedh, J. N. Andersen, On the origin of the Ru-$3d_{5/2}$ satellite feature from RuO_2(110). *Surf. Sci. Lett.* **504**, L196–L200 (2002).
